# NOX4 modulates breast cancer progression through cancer cell metabolic reprogramming and CD8^+^ T cell antitumor activity

**DOI:** 10.3389/fimmu.2025.1534936

**Published:** 2025-02-07

**Authors:** Yingying Xiong, Yiming Weng, Shan Zhu, Jian Qin, Jia Feng, Xiaopeng Jing, Chao Luo, Wei Gong, Rui Sun, Min Peng

**Affiliations:** ^1^ Department of Clinical Laboratory, Wuhan Fourth Hospital, Wuhan, China; ^2^ Cancer Center, Renmin Hospital, Wuhan University, Wuhan, China; ^3^ Department of Breast and Thyroid Surgery, Renmin Hospital of Wuhan University, Wuhan, China; ^4^ Central Laboratory, Renmin Hospital, Wuhan University, Wuhan, China; ^5^ Department of Central Laboratory, The Affiliated Huaian No.1 People’s Hospital, Nanjing Medical University, Huai’an, China; ^6^ Department of Oncology, XiangYang Central Hospital, Hubei University of Arts and Science, Xiangyang, China

**Keywords:** breast cancer, fatty acid oxidation, metabolic reprogramming, MYC, NOX4

## Abstract

**Introduction:**

Breast cancer is the most frequently diagnosed malignancy and a leading cause of cancer-related mortality among women worldwide. Although NADPH oxidase 4 (NOX4) has been implicated in various oncogenic processes, its exact function in breast cancer progression, metabolic reprogramming, and immune modulation remains unclear.

**Methods:**

We used murine 4T1 and EO771 breast cancer models to generate NOX4 knockout (KO) cell lines via CRISPR/Cas9. *In vitro* assays (cell proliferation, colony formation, wound healing, and Seahorse metabolic analyses) and *in vivo* orthotopic tumor studies assessed the impact of NOX4 loss. Transcriptomic changes were identified through RNA sequencing and gene set enrichment analysis. We performed MYC knockdown in NOX4 KO cells to investigate its mechanistic role. Flow cytometry characterized tumor-infiltrating immune cells. Finally, NOX4-overexpressing cells were tested for survival benefit and response to dual-checkpoint immunotherapy (anti-PD-1/anti-CTLA-4).

**Results:**

NOX4 deletion accelerated tumor growth *in vivo* and enhanced proliferation, colony formation, and migratory capacity *in vitro*. Metabolic profiling showed that NOX4 KO cells had elevated glycolysis and fatty acid oxidation, along with increased mitochondrial mass. Transcriptomic and enrichment analyses revealed MYC pathway activation in NOX4 KO cells; suppressing MYC reversed these hyperproliferative and metabolic changes. Immunologically, NOX4 KO reduced CD8+ T cell infiltration and function, partially due to lowered CCL11/CCL5 levels, while PD-L1 expression was upregulated. In contrast, NOX4 overexpression improved survival in mice and synergized with checkpoint blockade, demonstrating a positive effect on anti-tumor immunity.

**Discussion:**

These findings show that NOX4 constrains breast cancer aggressiveness by limiting MYC-driven metabolic adaptations and supporting CD8+ T cell-mediated immunity. Loss of NOX4 promotes a more malignant phenotype and dampens T cell responses, whereas its overexpression prolongs survival and enhances checkpoint inhibitor efficacy. Therapeutically targeting the NOX4–MYC axis and leveraging NOX4’s immunomodulatory capacity could offer promising strategies for breast cancer management.

## Introduction

Breast cancer remains the most diagnosed cancer and a leading cause of cancer-related deaths among women in the worldwide (1). Despite the significant advancements in prevention and treatment over the past few decades, the challenges of drug resistance and metastasis persist (2). This underscores the urgent need to delve into the mechanisms underlying the malignant behavior of breast cancer and to identify new therapeutic targets.

NADPH oxidases (NOX) are a family of enzymes that play a pivotal role in the production of reactive oxygen species (ROS). There are seven enzymes in the NOX family: NOX1-5 and dual oxidase (DUOX) 1–2. NOX enzymes in humans play important roles in diverse biological functions and vary in expression from tissue to tissue (3). Among the seven NOX isoforms, NOX4 is particularly significant due to its constitutive activity and ubiquitous expression (4), which are critical for various cellular processes, including signaling, differentiation, and host defense (4–6). However, in the context of cancer, NOX4’s role is more complex. NOX4 plays a complex and variable role in malignant tumors, showing different effects across various cancer types. NOX4 has been found to promotes tumor growth by being highly expressed in non-small cell lung cancer tissues, enhancing gastric cancer cell proliferation through the GLI1 pathway, and regulating TGF-β1–driven metabolic rewiring in glioblastoma cells (7–9). Conversely, in pancreatic cancer, NOX4 acts as a tumor suppressor by inducing reactive oxygen species-mediated cellular senescence (10), and NOX4 prevent HCC progression via regulating redox and metabolic homeostasis (11). In additional, it can also modulate the tumor microenvironment and immune response (12–14).

This complex nature of NOX4, acting both as a mediator of normal physiological functions and as a potential driver or suppressor of malignancy, makes it an intriguing subject for cancer research, particularly in understanding its contribution to breast cancer pathophysiology and therapy. The exact role of NOX4 in breast cancers is still not fully unveiled.

Studies have shown that NOX4 plays an important role in the metabolism and migration of breast cancer cells (15). Metabolic reprogramming is a fundamental hallmark of cancer (16). This metabolic reprogramming involves enhanced glycolysis and alters mitochondrial function, which supports the increased energy demands of rapidly proliferating tumor cells. Among the various forms of metabolic alterations, aerobic glycolysis is the most extensively studied. Unlike normal cells, which predominantly rely on oxidative phosphorylation for energy production, tumor cells preferentially utilize aerobic glycolysis. and the increased products of glycolysis can serve as raw materials for the synthesis of macromolecules that support tumor proliferation.

The relationship between NOX4 expression in breast cancer and its impact on treatment response and patient prognosis has been investigated in many studies, previous studies demonstrated that Myc signaling enhances the migration ability of breast cancer cells (17). The Myc signaling pathway is a critical regulator of cell growth and metabolism, and its activation is associated with the progression of various cancers. further studies (18) proposed the interaction mechanism between NOX4 and Myc, exploring how this interaction contributes to the aggressive behavior of breast cancer cells and suggesting potential therapeutic interventions targeting this pathway. We found that knockout of NOX4 enhances tumor growth in breast cancer models, suggesting a complex role of NOX4 in tumor dynamics that may depend on the context and specific cellular environment. Moreover, NOX4 knockout promote the malignant behavior of breast cancer cells and activates the Myc signaling pathway in these cells.

Since NOX4 is a key enzyme involved in the metabolism, our data showed that NOX4 knockout reshapes the metabolic profile of breast cancer cells, making their metabolism more invasive. This reshaping involves changes in both glycolytic and oxidative phosphorylation pathways, indicating that NOX4 may regulate a broad spectrum of metabolic processes directly or indirectly in cancer cells.

Further analysis indicated that Myc acts as an effector molecule mediating the increased malignancy in NOX4-deficient breast cancer cells. NOX4 also enhances the antitumor effect of CD8^+^ T cells. This was demonstrated by increased infiltration and activity of CD8^+^ T cells in NOX4 overexpressing tumors, T cell associated chemokines and tumor sourced PD-L1 might be involved. Suggesting that NOX4 may modulate the immune microenvironment to favor anti-tumor immunity. Through the overexpression of NOX4, we found that it can improve the prognosis of breast cancer and enhance the efficacy of tumor immunotherapy. The synergy between NOX4 overexpression and immunotherapeutic agents such as anti-PD-1 and anti-CTLA-4 antibodies suggests that targeting NOX4 could potentiate the effects of existing immunotherapies.

## Materials and methods

### Mice

C57BL/6 mice and Balb/c mice were purchased from the Model Animal Research Center of Nanjing University. All mice were housed in a specific pathogen-free facility in the central Laboratory Animal Center of Tongren hospital, Wuhan university. Animal experimental protocols were approved by the Review Committee of Wuhan University School of Medicine and were following institutional guidelines. Tumor onset was monitored by palpation and tumors were measured once a week using a caliper, and volume was calculated. Once tumors in the control group reached the maximum allowed size. Tumor burden was calculated by adding the volume or the weight of all the tumors from the same animal. For the orthotopic injection of murine E0771 breast cancer, (0.5×10^6^) EO771 or EO771 NOX4 KO, EO771NOX4 KO shMyc in 50 μL of PBS were injected into the fourth right mammary fat pad of anesthetized (with 4% isoflurane) 6-week-old female C57BL/6 mice, and the (0.2×10^6^) 4T1 or 4T1 NOX4 KO, 4T1 NOX4 KO shMyc cells in 50 μL of PBS were injected into the fourth right mammary fat pad of anesthetized (with 4% isoflurane) 6-week-old female Balb/C mice. In all mouse experiments, animals were monitored 3 times a week and tumor growth were measured using a caliper. Tumor volumes were calculated as follows: longer diameter × shorter diameter²/2. Animals were culled once tumors reached the maximum allowed size. Tumors were divided in portions for (a) preparation of cells isolation, tissue sections for H&E and IHC and (b). Studies were approved by the Wuhan University Institutional Animal Care and Use Committee.

### Cell culture

4T1 breast cancer cells and EO771 were purchased from the American Type Culture Collection. Parental or transfected cells were maintained in RPMI-1640 medium and DMEM medium (Mediatech, Inc.) containing 10% fetal bovine serum (FBS), penicillin (100 U/ml) and streptomycin (100 μg/ml). pX459 CRISPR/Cas9-Puro vector (Addgene, Cambridge, MA) was maintained in our lab.

### CRISPR/Cas9

Guide RNA (gRNA) sequences for CRISPR/Cas9 were designed using the CRISPR tool available at the Feng Zhang Lab website (http://crispr.mit.edu/). The oligonucleotide inserts for human NOX4 gRNA #1 and gRNA #2 were 5’-GCCAGGACTGTCCGGCACAT-3’ and 5’-AAGACTCTACACATCACATG-3’, respectively. These gRNAs were designed to target exon 3 and exon 4 of the NOX4 gene at chromosomal locations 87295825 and 87297513 on chromosome 7. Complementary oligonucleotides for the gRNAs were annealed and inserted into the pX459 CRISPR/Cas9-Puro vector. EO771 and 4T1 cells were transfected with either pX459/gRNA #1 or pX459/gRNA #2 using Lipofectamine 3000, following the manufacturer’s guidelines. After 48 hours, the cells were treated with 5 μg/ml of puromycin for 7 days to select for successfully transfected cells. Two weeks later, colonies were picked using cloning cylinders, and NOX4 gene editing was confirmed by T7 endonuclease I (T7E1) assay, DNA sequencing, and Western blot analysis.

#### Construction of NOX4 overexpression plasmid

The coding sequence of murine NOX4 was amplified by PCR using specific primers designed to include restriction enzyme sites for cloning. The PCR product was purified and digested with the appropriate restriction enzymes, followed by ligation into the pcDNA3.1(+) vector. The recombinant plasmid, pcDNA3.1(+)-NOX4, was verified by sequencing.

#### Stable transfection and selection of stable cell lines

4T1 and EO771 cells were transfected with the pcDNA3.1(+)-NOX4 plasmid using Lipofectamine 2000 (Invitrogen) according to the manufacturer’s instructions. Briefly, cells were seeded in 6-well plates at a density of 2x10^5^ cells/well and grown to 70-80% confluence. The DNA-Lipofectamine complexes were prepared by mixing 4 µg of plasmid DNA with 10 µL of Lipofectamine 2000 in 250 µL of Opti-MEM medium and incubated for 20 minutes at room temperature. The complexes were then added to the cells and incubated for 6 hours at 37°C. Following incubation, the medium was replaced with fresh DMEM supplemented with 10% FBS. Transfected 4T1 and EO771 cells were selected using G418 sulfate (Geneticin) at a concentration of 800 µg/mL. The selection medium was replaced every 3 days for 2 weeks. Individual colonies were picked and expanded in DMEM containing 400 µg/mL G418.

#### Gene expression analyses of clinical data sets and bioinformatics analyses

Associations between NOX4 mRNA expression and infiltration of different cell types from the TME were analyzed by using TIMER2.0 (PMID: 32442275) and TISIDB (http://cis.hku.hk/TISIDB/) [PMID:30903160].1,100 BRCA were incorporated. TISIDB was also used to analyze gene expression correlations. All correlations were calculated with Spearman’s rank correlation coefficient.

### Immunofluorescence

Cells were cultured in the Chamber Slide (Lab-Tek), cells were stained as the following procedure when the cells reached 60% confluence, 1) 10% methanol for 10 mins, 2) 0.1% TritonX100 5 mins, 3), blocked by 10% donkey serum at room temperature for 1 h, 4) incubated with rabbit anti human Ki67 (1:250), Alexa Fluor^®^ 488 labeled Tubulin (1:125) overnight at 4°C, primary antibodies were detected by Goat Anti-Rabbit IgG H&L (Alexa Fluor^®^ 594) (2ug/mL). Slides were counterstained with DAPI and images were captured on a Zeiss LSM 510 confocal microscope equipped with a digital image analysis system (Pixera).

#### Detection of ROS using DCF-DA

Parental cells and knock-out cells (1×10^4^) cells were seeded in 96 wells black plates with transparent bottom (Costar, Corning, NY, USA), cells were treated with TGF-β (5ng/ml,8 h)with/without VAS-2870 (2 µM,1 h), cells were washes for two times and then cells were incubated for 4 hr with 20 μM DCF-DA (Sigma Aldrich) which is activated by ROS to generate a highly fluorescent 2′7′-dichlorofluorescein (DCF) molecule. DCF fluorescence was measured at 485/530 nm using a Synergy H4 multimode plate reader (BioTek). Considering the different rate of cell proliferation, another parallel hole was used to count cells, and the number of cells was used to standardize the ROS level.

### Western blotting

The cells were harvested and lysed in RIPA buffer (50mM Tris-HCl pH 7.5, 150mM NaCl, 1% IPEGAL, 0.5% deoxycholate, 5mM EDTA) containing a protease inhibitor cocktail (Roche, Mannheim, Germany). For protein immunoblot analysis, polypeptides in whole cell lysates were resolved by SDS-PAGE and transferred to nitrocellulose membranes. The images were acquired using Odyssey (Licor bioscience, Lincoln, NE). The antibody for NOX4 (14347-1-AP), Myc (9E10)was purchased from proteintech and.Thermofisher.

#### Colony formation assay

Colony formation assays were performed following a previously established protocol with slight modifications (19). In brief, EO771 and 4T1 cells (100 per well) were plated in triplicate in six-well plates containing complete culture medium. After 10 days of incubation, the cells were fixed using methanol and stained with Crystal Violet. Colonies containing more than 50 cells were manually counted. Statistical differences between groups were evaluated using the Mann-Whitney nonparametric test for pairwise comparisons or one-way ANOVA with a Bonferroni *post-hoc* test for multiple group comparisons. A p-value of less than 0.05 was considered statistically significant.

#### Cell proliferation assay

Cells were suspended in DMEM or RPMI 1640 medium containing 10% FBS, 500 cells were seeded in 96-well plates, and then incubated for 1-5 days, cells were digested by trypsin and resuspended in 200 uL medium, OD600 was detected immediately, (BioTek, Winooski, VT).

### Preparation of tumor-infiltrating cells

Tumor-infiltrating cells were harvested from subcutaneous tumors at designated time points for further analysis. Tumor tissues were excised from euthanized mice and initially subjected to mechanical dissociation using sterile scalpels to break down the tissue into small pieces. These fragments were then enzymatically digested in a solution containing collagenase I (Worthington Biochemical, LS004197) and DNase I (1 mg/ml; Roche, 11284932001) at 37°C for 45 minutes to facilitate the breakdown of the extracellular matrix and release of infiltrating immune cells. During the digestion process, the tissues were gently agitated to ensure thorough enzymatic exposure and uniform digestion.

After digestion, 0.5 M EDTA was added for 5 minutes at 37°C to prevent the clumping of dendritic cells (DCs) and T cells, which can occur due to cell adhesion molecules and remaining tissue fragments. Following this, the cell suspension was passed through a 70-μm nylon cell strainer to remove any undigested tissue fragments and ensure a single-cell suspension. The filtered cell suspension was then centrifuged at 300 x g for 10 minutes at 4°C to pellet the cells, after which the supernatant was discarded, and the cell pellet was resuspended in cold PBS or a suitable buffer for further assays.

### Oxygen-consumption rate and extracellular acidification rate

EO771, EO771 NOX4 KO, EO771 NOX4 KO shMyc, 4T1, or 4T1 NOX4 KO, along with 4T1 NOX4 KO shMyc breast cancer cells, were seeded into XF-96 cell culture plates at a density of 0.2 × 10^5^ cells per well. The cells were cultured for 4 hours to ensure attachment to the plate. Following this, the cells were washed and incubated for 1 hour in XF assay medium (unbuffered DMEM, pH 7.4, with 2 mM L-glutamine, 10 mM glucose, and 2 mM sodium pyruvate for oxygen consumption rate (OCR) measurements, or glucose- and pyruvate-free medium for extracellular acidification rate (ECAR) assessment during the glycolysis stress test). This was performed in a non-CO_2_ incubator at 37°C according to the Seahorse Bioscience protocol. Real-time measurements of ECAR and OCR were carried out using an XF-96 Extracellular Flux Analyzer (Seahorse Bioscience). Three or more consecutive readings were taken under baseline conditions, followed by the sequential addition of 1.5 μM oligomycin to block mitochondrial ATP synthase; 5μM FCCP (fluoro-carbonyl cyanide phenylhydrazone) to uncouple ATP synthesis from oxygen consumption; and 0.5 μM each of rotenone and antimycin A to inhibit the electron transport chain. For glycolysis evaluation, three or more ECAR measurements were taken under basal conditions, followed by the sequential addition of 1.5 μM oligomycin to stimulate maximal glycolytic activity and 50 mM 2-deoxyglucose (2-DG) to inhibit glycolysis-driven ECAR.

### Reagents, antibodies, and flow cytometry

22 days post-inoculation, tumor-infiltrating lymphocytes (TILs) isolated from the tumors were initially incubated with anti-CD16/32 antibodies (Catalog# 14-0161-85, ThermoFisher Scientific) to block nonspecific antibody binding. Following this, cells were stained with the appropriate surface antibodies for 30 minutes at 4°C. After staining, the cells were washed with PBS containing 2% FCS (fetal calf serum) and prepared for flow cytometry (FACS) analysis. Tumor cells underwent two washes in preparation for FACS analysis.

For intracellular staining of the transcription factor Foxp3, the Foxp3 Fix/Perm Buffer Set (eBioscience, Thermo Fisher) was used according to the manufacturer’s protocol. To assess intracellular cytokines, cells were first stimulated for 4 hours with 50 ng/ml PMA and 1 µg/ml ionomycin in the presence of Brefeldin A (5 µg/ml; all reagents from Sigma). After stimulation, cells were stained for surface markers, followed by fixation and permeabilization using the Foxp3 Fix/Perm Buffer Set for intracellular cytokine detection.

The following antibodies were applied at dilutions ranging from 1/200 to 1/600: PerCP-Cy5.5-labeled anti-IL-17A (TC11-18H10.1), PE-labeled anti-IFN-γ (XMG1.2), APC-labeled anti-Foxp3 (FJK-16s, eBioscience, Thermo Fisher), FITC-labeled anti-CD11b (M1/70), and PE-, FITC-, or APC-labeled anti-CD4 (RM4-5). Additionally, FITC-, PerCP-Cy5.5-, or Pacific Blue-labeled anti-CD45 (30-F11), FITC-labeled anti-Ki67 (SolA15), FITC-labeled anti-Myc (9E10), and FITC-labeled anti-PD-L1 (MIH5) were used. All antibodies were sourced from ThermoFisher unless otherwise specified. Flow cytometry data were acquired using a 5-color FACScan (Becton Dickinson) and analyzed with FlowJo software (Treestar).

### Cytokine analysis

The quantity of CCL11, CCL5 (Bosterbio) were determined in tumor tissue using ELISA kits according to the manufacturer’s instructions. Tumors harvested after 22 days of growth were processed by homogenizing 1g of tissue in 2mL of tissue lysis buffer. Following homogenization, the samples were centrifuged to remove the precipitate. Supernatant was collected and utilized for subsequent analyses. The sensitivity of the assays was <20 pg/ml.

### Wound healing assay

4T1 or EO771 breast cancer cells were cultured in 6-well plates until they reached 90-100% confluence. A linear scratch was made across the cell monolayer using a sterile 200 µL pipette tip. The cells were washed with phosphate-buffered saline (PBS) to remove detached cells and then cultured in serum-free medium. Images of the wound were taken immediately after scratching (0 hours) and 24 hours later using an inverted microscope. The wound area was measured with ImageJ software, and the migration rate was calculated as the percentage of wound closure over 24 hours. All experiments were performed in triplicate.

### RNA extraction and PCR

Total RNA was extracted from tumor cells using the Qiagen RNeasy RNA isolation kit, and this RNA was then used for cDNA synthesis. A total of 1 μg of RNA was reverse transcribed with Superscript IV and random primers (Invitrogen). To quantify the expression of target genes, cDNA samples were amplified on an Applied Biosystems Realtime PCR system with SYBR Green Master Mix (Invitrogen) and gene-specific primers (listed in **Supplementary Table S1**), following the manufacturer’s protocol. The relative changes in mRNA expression between experimental and control groups were calculated using the ^ΔΔ^CT method. β-actin mRNA levels were used to normalize the results for each sample, and expression levels were presented as fold changes over baseline, with baseline set to 1. Statistical differences between groups were assessed using a two-sided Student’s t-test for pairwise comparisons or one-way ANOVA for multiple groups. Error bars on graphs represent the mean ± SEM unless otherwise indicated. All primers were obtained from Thermo Fisher Scientific.

### Assessment of mitochondrial morphology

The following dyes, purchased from Invitrogen, were used for staining as per the manufacturer’s guidelines: MitoTracker Green FM and tetramethylrhodamine ethyl ester perchlorate (TMRE). Mitochondrial membrane potential was measured using 100 nM TMRE, following the manufacturer’s protocol. Total mitochondrial mass was assessed using MitoTracker Green, also following the provided instructions. After staining, all cells were analyzed by flow cytometry. For imaging, cells were mounted on poly-L-lysine coated slides. Confocal microscopy images were captured using a Zeiss LSM 800 microscope and processed with Zen 2.3 software (Zeiss). Image analysis was performed using the Pixera digital image analysis system.

### Gene expression profiling by RNA-seq

Tumor cells in the logarithmic growth phase, with approximately 70% confluence, were harvested 24 hours post-passage, and total RNA was extracted for RNA sequencing. A Poly(A) RNA sequencing library was prepared using Illumina’s TruSeq Stranded mRNA sample preparation protocol. RNA integrity was assessed using the Agilent Technologies 2100 Bioanalyzer. Paired-end sequencing was performed on the Illumina NovaSeq 6000 system. Before assembly, reads containing sequencing adapters, primers, or low-quality sequences (with a quality score below 20) were filtered out. The cleaned reads were then aligned to the reference genome using the HISAT2 package, allowing up to 20 alignments per read and permitting a maximum of two mismatches. HISAT2 also generated a database of potential splice junctions for accurate alignment. The aligned reads for each sample were assembled into transcripts using StringTie, and transcriptomes from all samples were combined to create a comprehensive transcriptome using a proprietary Perl script developed by LC Sciences (Houston, Texas, USA). After transcriptome assembly, FPKM values were calculated by StringTie, and differential gene expression was analyzed using edgeR. Genes with log2(fold change) ≥1 or ≤-1 and p-values < 0.05 were considered differentially expressed.

### Quantification and statistical analysis

Results are presented as Mean ± SEM unless stated otherwise. For continuous variables, t-tests were utilized. The log-rank test was applied to assess survival differences between groups. Statistical significance is indicated by asterisks, with * representing p < 0.05, ** indicating p < 0.01, and *** denoting p < 0.001. The symbol ‘n’ denotes the number of independent samples.

## Results

### Knockout of NOX4 enhances tumor growth in breast cancer models

To investigate the role of NOX4 in breast cancer, firstly, we generated two NOX4 knockdown cell lines, EO771 NOX4 KO and 4T1 NOX4 KO. The CRISPR-Cas9 system was employed to target specific sequences within the NOX4 gene, resulting in the disruption of its normal function. Single guide RNAs (sgRNAs) were designed to direct Cas9 endonuclease to exon 3 and exon 4 of the target cells. Following transfection, cells were selected and expanded to establish stable knockout cell lines.

The identity and successful knockout of these cell lines were confirmed via western blotting and DNA sequencing. Western blot analysis revealed the absence of NOX4 protein in both EO771 NOX4 KO and 4T1 NOX4 KO cells, indicating successful knockout at the protein level (**Supplementary Figure S1A**). DNA sequencing further validated the gene disruption, showing a deletion of six bases in exon 3 in EO771 NOX4 KO cells and a six-base mismatch in exon 4 in 4T1 NOX4 KO cells (**Supplementary Figure S1B**), consistent with the CRISPR-Cas9 design.

To further evaluate the functional impact of NOX4 knockout, we examined ROS production in the presence and absence of VAS-2870, a specific NOX4 inhibitor (20). Cells were treated with TGF-β, a known inducer of ROS generation (21), and ROS levels were measured. In control cells, TGF-β treatment significantly increased ROS production, which was effectively reduced by VAS-2870 treatment. However, in NOX4 KO cells, TGF-β-induced ROS production was minimal and unaffected by VAS-2870, confirming the successful and functional knockout of NOX4 in these cell lines (**Supplementary Figure S1C**).

**Figure 1:**
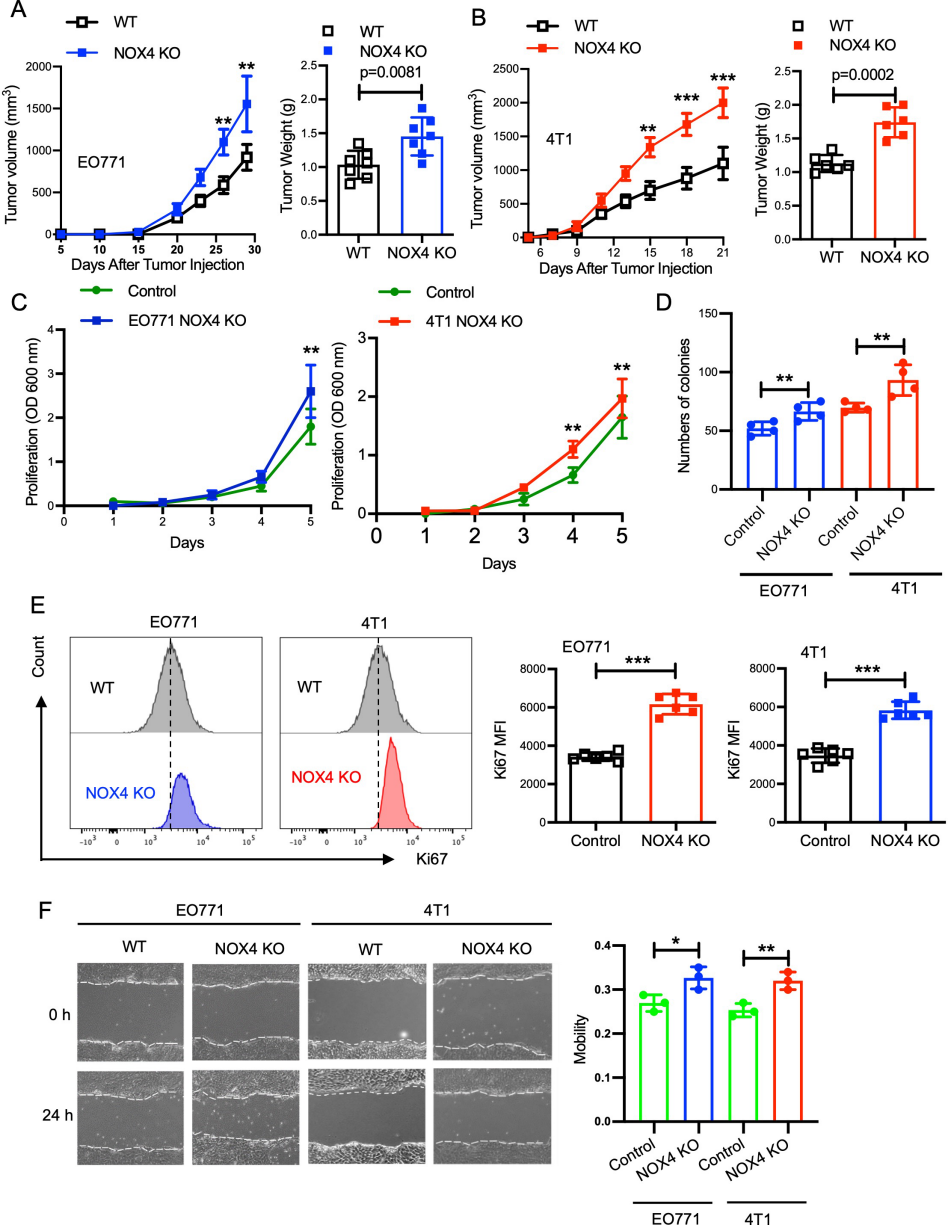
Knockout of NOX4 Enhances Tumor Growth in Breast Cancer Models. **(A)** Tumor volume measured over time in EO771 NOX4 wild-type (WT) and NOX4 KO cell lines (left). Tumor weight at the endpoint for EO771 NOX4 WT and NOX4 KO cell lines (right). N=7 per group. **(B)** Tumor volume measured over time in 4T1 NOX4 WT and NOX4 KO cell lines (left). Tumor weight at the endpoint for 4T1 NOX4 WT and NOX4 KO cell lines (right). N=6 per group. **(C)** Growth curves of EO771 and 4T1 NOX4 control and NOX4 knockout (KO) cells over a period of time. Data is pooled from three independent experiments. **(D)** Quantification of colony formation in EO771 and 4T1 NOX4 WT and NOX4 KO cells. **(E)** Representative histograms showing Ki67 expression in EO771 and 4T1 NOX4 wild-type (WT) and NOX4 knockout (KO) cells. Quantification of Ki67 Mean Fluorescence Intensity (MFI): Bar graphs displaying the MFI of Ki67 in EO771 and 4T1 NOX4 WT and NOX4 KO cells. ***p<0.001. **(F)** Wound healing assay was used to evaluation of the migration ability of EO771 and 4T1 NOX4 WT and NOX4 KO cells, representative images of wound closure at 0 and 24 hours for EO771 and 4T1 NOX4 WT and NOX4 KO cells. Quantification of wound closure. Data is pooled from three independent experiments and presented as mean ± SEM, a two-tailed Student’s t-test is used for the statistical analysis. Scale bars in confocal images represent 50 μm. *p<0.05, **p<0.01.

To investigate the role of NOX4 in breast cancer development, we conducted *in vivo* experiments using mouse models. Control and NOX4 KO EO771 and 4T1 cells were inoculated into the right inguinal mammary fat pad of B6 and Balb/C mice, respectively. Tumor growth was monitored over a defined period, with measurements taken at regular intervals to assess tumor volume and progression. The results demonstrated that tumors derived from NOX4 KO cells grew significantly faster and resulted in heavier tumor weights compared to those from control cells. This trend was consistent in both EO771 and 4T1 NOX4 KO groups (**Figures 1A, B**; **Supplementary Figure S1D**). The enhanced tumor growth in NOX4 KO groups suggests that NOX4 acts as a tumor suppressor in these breast cancer models. To further elucidate the role of NOX4 in breast cancer cells, in cell proliferation assay, we observed a significant increase in the proliferation rate of NOX4 knockout (KO) cells compared to wild-type (WT) cells. Specifically, NOX4 KO cells exhibited a steeper proliferation curve, indicating a faster doubling time (**Figure 1C**). This was further corroborated by clonal formation assays, which demonstrated a higher number of cell clones in NOX4 KO cells (**Figure 1D**).

Given the pivotal role of Ki67 as a marker for cell proliferation, we employed intracellular Ki67 flow cytometry analysis to quantify the proliferation rates more precisely. This analysis indicated a markedly higher expression of Ki67 in NOX4 KO cells relative to control cells, with an increase of approximately 30% in Ki67-positive cells (**Figure 1E**). This trend was consistently observed in both EO771 and 4T1 cell lines, reinforcing the generalizability of our findings across different breast cancer models. To further validate these observations, we performed immunofluorescence analysis, which also confirmed elevated Ki67 expression in NOX4-KO cells when compared to control cells. The immunofluorescence staining showed a significant increase in the intensity and frequency of Ki67-positive nuclei in NOX4 KO cells (**Supplementary Figure S1E**). This suggests that the absence of NOX4 promotes a higher proliferation rate at the single-cell level, contributing to the overall increase in cell proliferation observed in the assays. The scratch assay was used to evaluate the migration ability of cells after NOX4 knockout, and the results indicated that NOX4 knockout led to enhanced cell migration ability (**Figure 1F**). These results collectively suggest that NOX4 deletion enhances proliferative capacity, migration ability in breast cancer cells, NOX4 knockdown act as a malignant behaviors promotor in breast cancers.

### NOX4 knockout reshaped the metabolic profile of breast cancer cells

**Figure 2:**
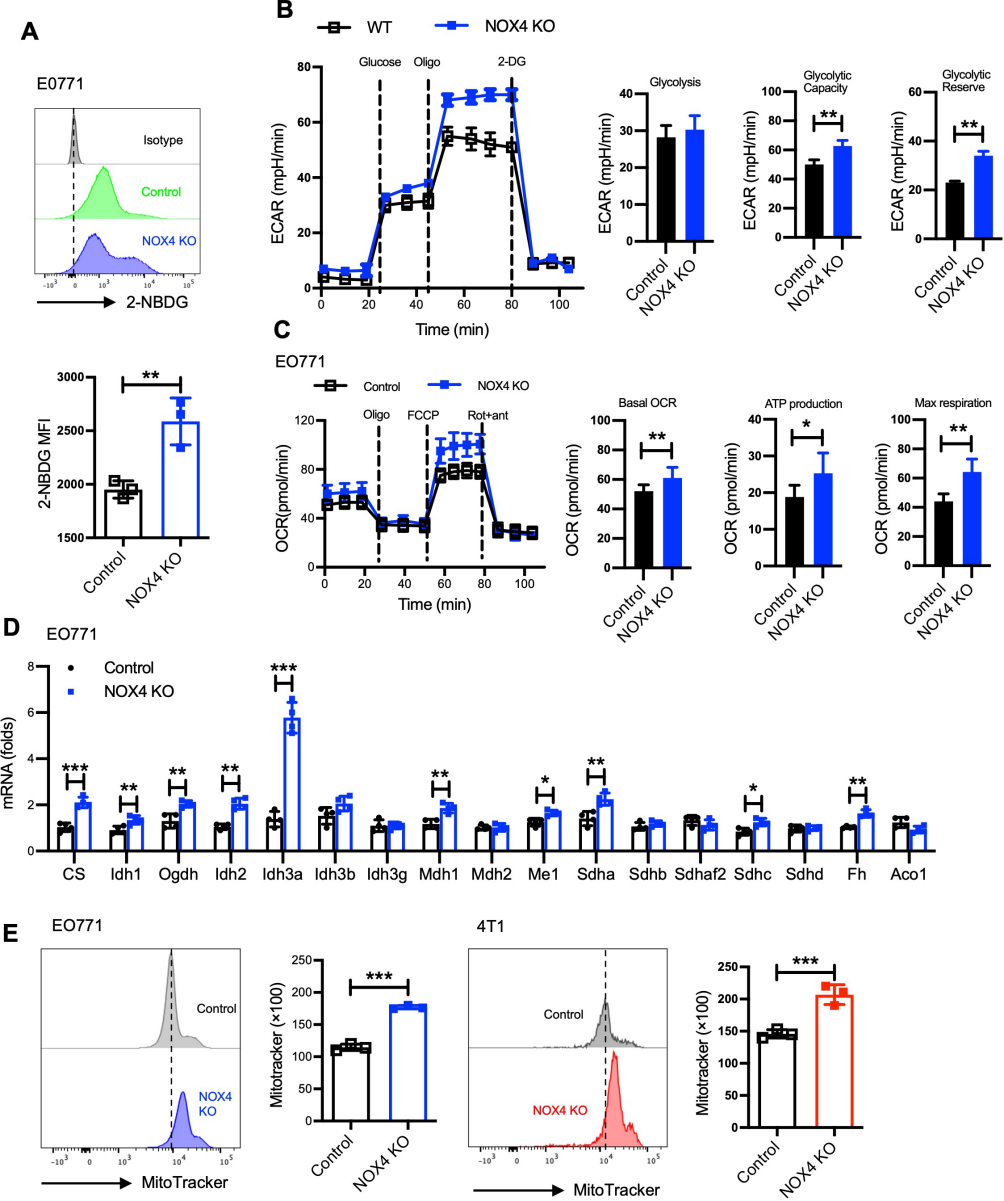
NOX4 knockout reshaped the metabolic profile of breast cancer cells. **(A)** Flow cytometry histograms showing 2-NBDG uptake in EO771 NOX4 wild-type (Control) and NOX4 knockout (KO) cells. Bar graph quantifying the mean fluorescence intensity (MFI) of 2-NBDG, Data are presented as mean ± SEM from 3 independent experiments.**p<0.01. **(B)** Extracellular acidification rate (ECAR) measurements in EO771 NOX4 WT and NOX4 KO cells over time, following the addition of glucose, oligomycin (Oligo), and 2-deoxyglucose (2-DG). Bar graphs quantifying glycolysis, glycolytic capacity, and glycolytic reserve, Data are presented as mean ± SEM from 3 independent experiments.**p<0.01. **(C)** Oxygen consumption rate (OCR) measurements in EO771 NOX4 WT and NOX4 KO cells over time, following the addition of oligomycin (Oligo), FCCP, and rotenone/antimycin A (Rot+ant). Bar graphs quantifying basal OCR, ATP production, and maximal respiration, indicating significant metabolic changes in NOX4 KO cells compared to controls, Data are presented as mean ± SEM from 3 independent experiments.*p<0.05, **p<0.01. **(D)** The relative mRNA expression levels of various TCA cycle genes in EO771 NOX4 WT and NOX4 KO cells. Data are presented as mean ± SEM from 4 independent experiments. *p<0.05, **p<0.01, ***p<0.001. **(E)** Flow cytometry histograms of MitoTracker staining in EO771 and 4T1 NOX4 WT (Control) and NOX4 KO cells. Bar graphs quantifying MitoTracker fluorescence intensity, Data are presented as mean ± SEM from 3 independent experiments.***p<0.001. Statistical significance was determined using a two-tailed Student’s t-test.

Adapting to challenging environments by altering their metabolic pathways is a critical mechanism for the sustained survival of tumor cells. To investigate the impact of NOX4 knockout on glucose metabolism in breast cancer cells, we conducted a series of experiments utilizing EO771 and 4T1 cell lines. Glucose uptake is dependent on a family of eight developmentally regulated glucose transporters, each of which has a specific tissue distribution, it’s the first step for glucose utilize *in vivo*. Initially, we examined glucose uptake in NOX4 knockout (KO) and control EO771 cells using 2-NBDG, a fluorescent glucose analog. Similar to glucose, 2-NBDG is taken up by cells via glucose transporters, and the post-uptake signal intensity reflects the level of glucose intake. Our results demonstrated that NOX4 KO EO771 cells exhibited significantly higher 2-NBDG uptake compared to control cells (**Figure 2A**), indicating an increased glucose uptake capability.

We then utilized the Seahorse XF Analyzer to measure the extracellular acidification rate (ECAR) as an indicator of glycolytic activity. The findings revealed that NOX4 KO cells possess higher glycolytic capacity and glycolytic reserve than control cells (**Figure 2B**). This increase in glycolytic function was also observed in 4T1 cells, suggesting a consistent trend across different breast cancer cell lines (**Supplementary Figure S2B**).

Given that glucose and fatty acid metabolism are major sources of cellular energy, we further assessed the fatty acid oxidation (FAO) capacity using the Seahorse XF Analyzer. The results indicated that NOX4 knockout led to increased basal oxygen consumption rate (OCR), ATP production, and maximum respiration in both EO771 and 4T1 cells (**Figure 2C**; **Supplementary Figure S2C**). These observations suggest that NOX4 knockout enhances the fatty acid metabolic capacity of the cells.

The tricarboxylic acid (TCA) cycle is a crucial metabolic pathway for energy production within cells. We employed real-time PCR to quantify the expression levels of key enzymes involved in the TCA cycle. The results showed a significant upregulation of these enzymes in NOX4 KO cells, indicating an enhancement of the TCA cycle (**Figure 2D**). Furthermore, mitochondrial analysis using MitoTracker staining revealed that NOX4 KO cells have larger mitochondria compared to WT cells (**Figure 2E**). This increase in mitochondrial mass provides a structural basis for the enhanced energy metabolic state observed in NOX4 KO cells.

These findings underscore the pivotal role of NOX4 in regulating metabolic pathways crucial for tumor cell survival. The enhanced glycolytic and fatty acid metabolic capacities, along with the upregulation of the TCA cycle and increased mitochondrial mass, highlight the metabolic adaptability of NOX4 KO cells. This metabolic reprogramming not only supports tumor growth but also presents potential therapeutic targets. Targeting NOX4 or its downstream metabolic pathways could alter the metabolic landscape of breast cancer cells.

### NOX4 knockout activated the Myc signaling pathway in breast cancer cells

**Figure 3:**
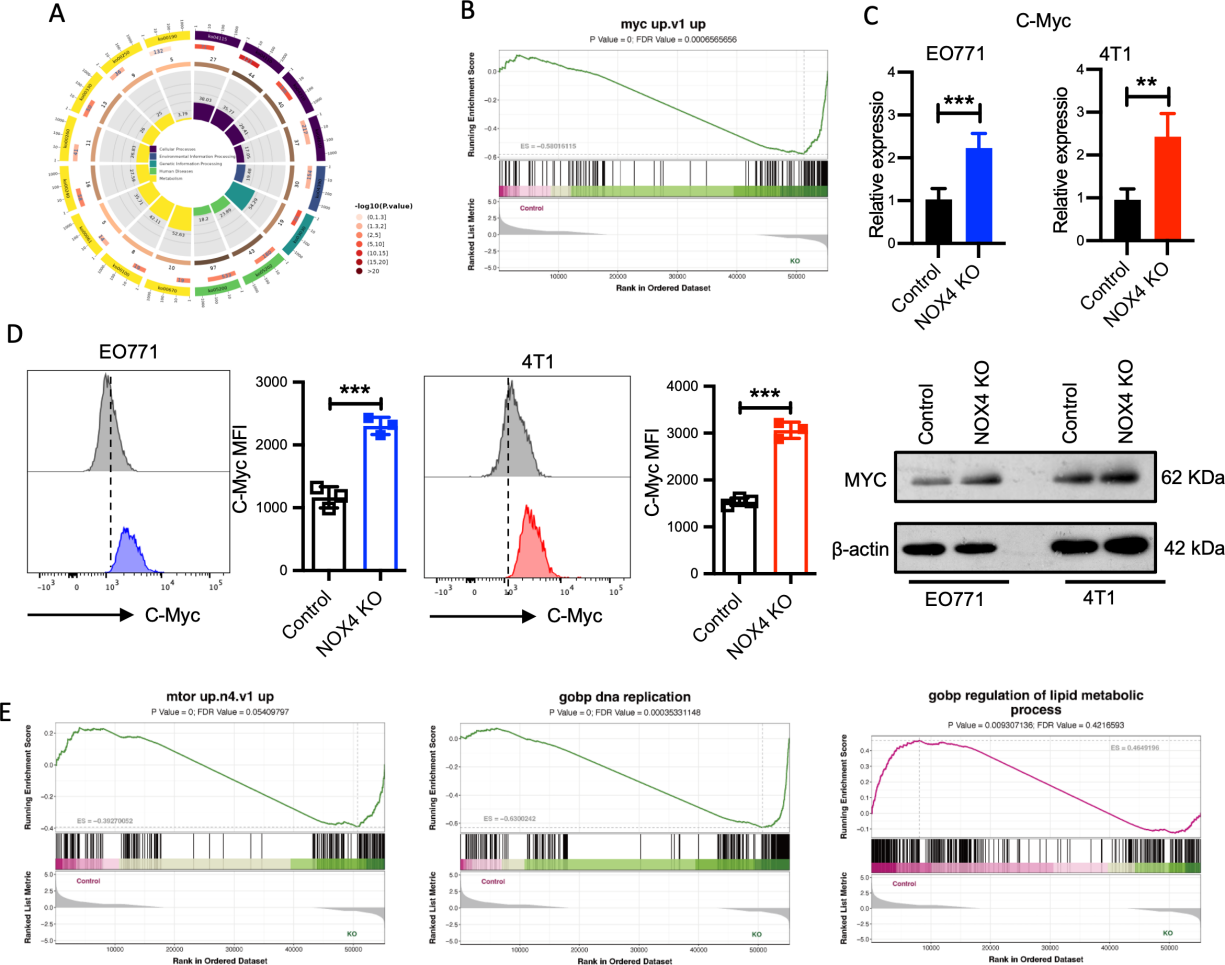
Transcriptomic and Functional Analysis of NOX4 Knockout Cells **(A)** KEGG Pathway Enrichment Analysis: Circular plot representing the KEGG pathway enrichment of differentially expressed genes in NOX4 knockout cells. Pathways are colored based on -log10(p-value), indicating the significance of enrichment. **(B)** GSEA of the "myc up.v1" gene set in NOX4 knockout cells. **(C)** mRNA expression levels of Myc genes in EO771 and 4T1 NOX4 wild-type (WT) and NOX4 knockout (KO) cells. **(D)** Flow Cytometry Analysis of MYC Expression. Histograms showing MYC protein expression in EO771 WT (grey) and NOX4 KO (blue) cells. Histograms showing MYC protein expression in 4T1 WT (grey) and NOX4 KO (red) cells. And western blotting analysis of MYC (right panel). ***p<0.001. **(E)** GSEA Enrichment of Different Pathways: GSEA plot for "mtor up.n4.v1" pathway, "DNA replication" pathway, “regulation of lipid metabolic process" pathway. Data are presented as mean ± SEM from 3 independent experiments. Statistical significance was determined using two-tailed Student’s t-test. **p<0.01.

To investigate the mechanisms underlying the altered metaboic reprogramming,and malignant potential in breast cancer associated with NOX4, we selected EO771 cells as a representative model and performed RNA sequencing on control and NOX4 knockout (KO) cells. Comprehensive transcriptomic analysis revealed extensive alterations in gene expression profiles between the control and NOX4 KO cells. Gene Ontology (GO) pathway enrichment analysis of the differentially expressed genes highlighted significant enrichment in tumor-related pathways, notably DNA replication, one-carbon pool by folate, and various metabolic pathways (**Figure 3A**; **Supplementary Figure S3A**).

To delve deeper into the functional implications of these transcriptomic changes, we conducted Gene Set Enrichment Analysis (GSEA) on the statistically significant genes. The GSEA results indicated a marked activation of the Myc signaling pathway(**Figure 3B**), a critical regulator of cell proliferation, apoptosis, and metabolism (22). To validate these findings at the transcriptional level, we performed quantitative PCR (qPCR) to measure *myc* mRNA expression in both EO771 and 4T1 cell lines (control and NOX4 KO). The qPCR results demonstrated a significant upregulation of *myc* mRNA in NOX4 KO cells compared to WT cells (**Figure 3C**), with consistent observations in both cell lines, suggesting a robust regulatory effect of NOX4 on *myc* expression.

To further corroborate the transcriptional data, we conducted flow cytometry analysis to quantify intracellular MYC protein levels. The flow cytometry and western blotting results agreed with the qPCR findings, showing a substantial increase in MYC protein expression in NOX4 KO cells (**Figure 3D**). This elevation in MYC protein levels underscores the potential role of MYC in mediating the enhanced proliferative and malignant phenotype observed in NOX4-deficient breast cancer cells. GSEA indicated significant enrichment of the mTOR signaling pathway, along with pathways related to DNA replication and lipid metabolic processes (**Figure 3E**). The VEGF pathway, which is crucial for angiogenesis and tumor growth, was also enriched (**Supplementary Figure S3B**). The activation of these pathways, particularly the mTOR and VEGF pathways, is strongly associated with increased malignancy and aggressive tumor behavior. These findings collectively suggest that NOX4 deletion significantly enhances the malignant potential of breast cancer cells by modulating key oncogenic signaling pathways, MYC and mTOR might be included, which are essential for tumor cell proliferation, survival, and metabolic reprogramming.

### Myc act as an effector molecule mediating increased malignancy in NOX4-deficient breast cancer cells

**Figure 4:**
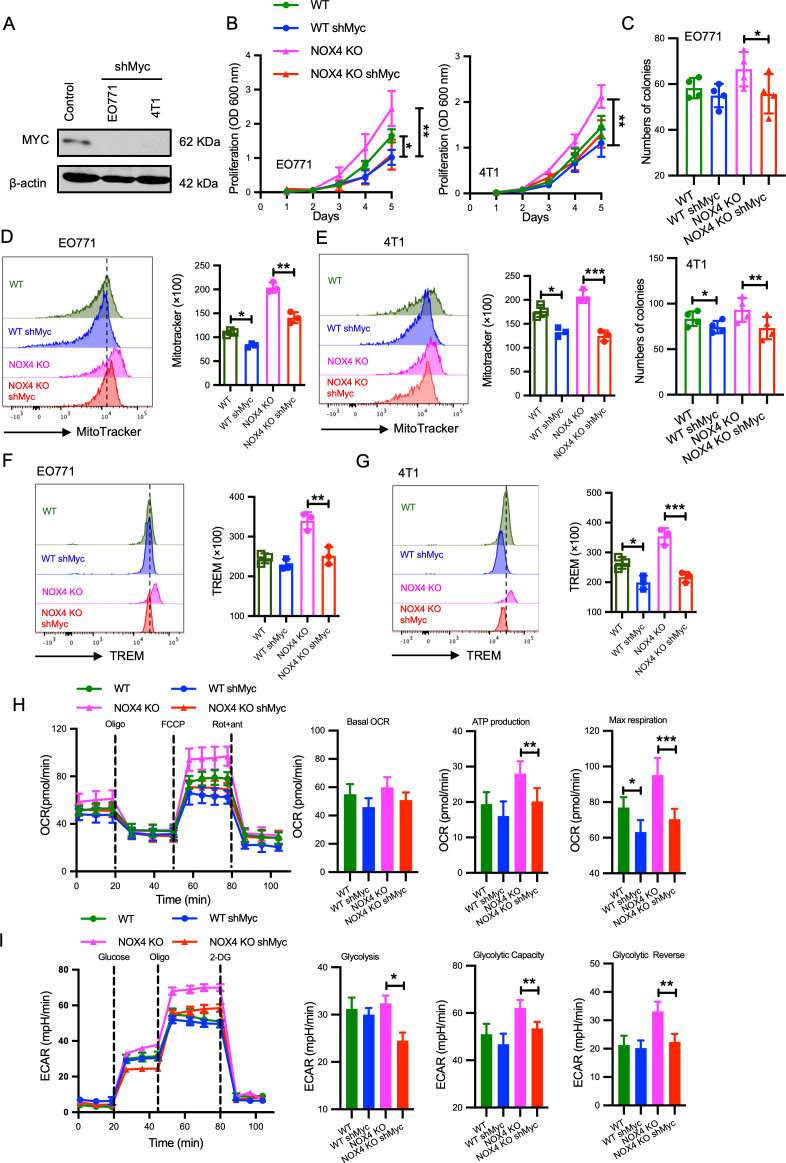
Myc as an Effector Molecule Mediating Increased Malignancy in NOX4-Deficient Breast Cancer Cells **(A)** MYC protein levels was detected by Western Blot in EO771 and 4T1 cells with and without MYC knockdown (shMyc). β-actin is used as control. **(B)** Growth curves of EO771 and 4T1 NOX4 knockout (KO) cells with and without MYC knockdown over several days. **p<0.01. **(C)** Quantification of colony formation in EO771 and 4T1 NOX4 KO cells with and without MYC knockdown. *p<0.05, **p<0.01. **(D)** Flow cytometry histograms showing mitochondrial mass in EO771 NOX4 KO cells with and without MYC knockdown. Bar graph quantifying MitoTracker fluorescence intensity **p<0.01. **(E)** Flow cytometry histograms showing mitochondrial mass in 4T1 NOX4 KO cells with and without MYC knockdown. Bar graph quantifying MitoTracker fluorescence intensity, *p<0.05. **(F)** Flow cytometry histograms showing TREM levels in EO771 NOX4 KO cells with and without MYC knockdown. Bar graph quantifying TREM levels**p<0.01. **(G)** Flow cytometry histograms showing TREM levels in 4T1 NOX4 KO cells with and without MYC knockdown. Bar graph quantifying TREM levels, ***p<0.001. **(H)** Oxygen consumption rate (OCR) measurements in EO771 NOX4 KO cells with and without MYC knockdown over time, following the addition of oligomycin (Oligo), FCCP, and rotenone/antimycin A (Rot+ant). Bar graphs quantifying basal OCR, ATP production, and maximal respiration, *p<0.05, **p<0.01, ***p<0.001. **(I)** Extracellular acidification rate (ECAR) measurements in EO771 NOX4 KO cells with and without MYC knockdown over time, following the addition of glucose, oligomycin (Oligo), and 2-deoxyglucose (2-DG). Bar graphs quantifying glycolysis, glycolytic capacity, and glycolytic reserve *p<0.05, **p<0.01. Data are presented as mean ± SEM from 3 independent experiments. Statistical significance was determined using two-tailed Student’s t-test.

To elucidate the role of Myc in the enhanced malignancy observed in NOX4-deficient breast cancer cells, we established EO771 and 4T1 cell lines with concurrent NOX4 knockout and MYC knockdown (**Figure 4A**). Comparative analyses of cell proliferation between control groups and Myc knockdown cells revealed that MYC knockdown significantly attenuated the increased cell proliferation induced by NOX4 knockout, with consistent findings across both EO771 and 4T1 cell lines (**Figure 4B**). Clonal formation assays further corroborated these results, indicating a significant reduction in colony formation in MYC knockdown cells compared to NOX4 knockout cells (**Figure 4C**).

Given the critical role of Myc signaling in the regulation of cellular metabolism (22), we assessed the impact of Myc on mitochondrial function by measuring mitochondrial mass, MitoTracker, an indicator of mitochondrial mass was used. Knockdown of Myc resulted in a noticeable reduction in mitochondrial mass in both EO771 NOX4 KO and 4T1 NOX4 KO cells (**Figures 4D, E**). Furthermore, TREM staining, which evaluates mitochondrial membrane potential, showed a significant decrease in membrane potential levels following Myc knockdown (**Figures 4F, G**).

Previous results indicated that fatty acid oxidation (FAO) levels were enhanced in NOX4-deficient breast cancer cells (**Figures 2C**; **Supplementary Figure S2C**). Considering the observed reduction in mitochondrial mass and membrane potential upon Myc knockdown. Seahorse assay revealed that Myc knockdown mitigated the enhanced energy metabolism induced by NOX4 knockout (**Figure 4H**). Specifically, ATP production, maximal respiration was all decreased in Myc knockdown cells (**Figure 4H**). Additionally, reductions in glycolysis, glycolytic capacity, and glycolytic reserve were observed compared to control groups (**Figure 4I**). These results collectively suggest that Myc mediates the increased malignancy in NOX4-deficient breast cancer cells. Inhibiting Myc signaling can reverse the abnormal energy metabolism and reduce the proliferative capacity induced by NOX4 knockout, highlighting the key effect of Myc in NOX4-related breast cancer malignancy.

### NOX4 enhanced the CD8^+^ T cells mediate antitumor effect

Breast cancer is characterized by a complex immune microenvironment (23). The tumor microenvironment (TME) plays a crucial role in tumor behavior and treatment response, making its pathologic assessment essential for disease management. The interaction between cancer cells and their microenvironment significantly influences their response to therapies, especially on the modification of CD8^+^ mediate anti-tumor reaction (24).

**Figure 5:**
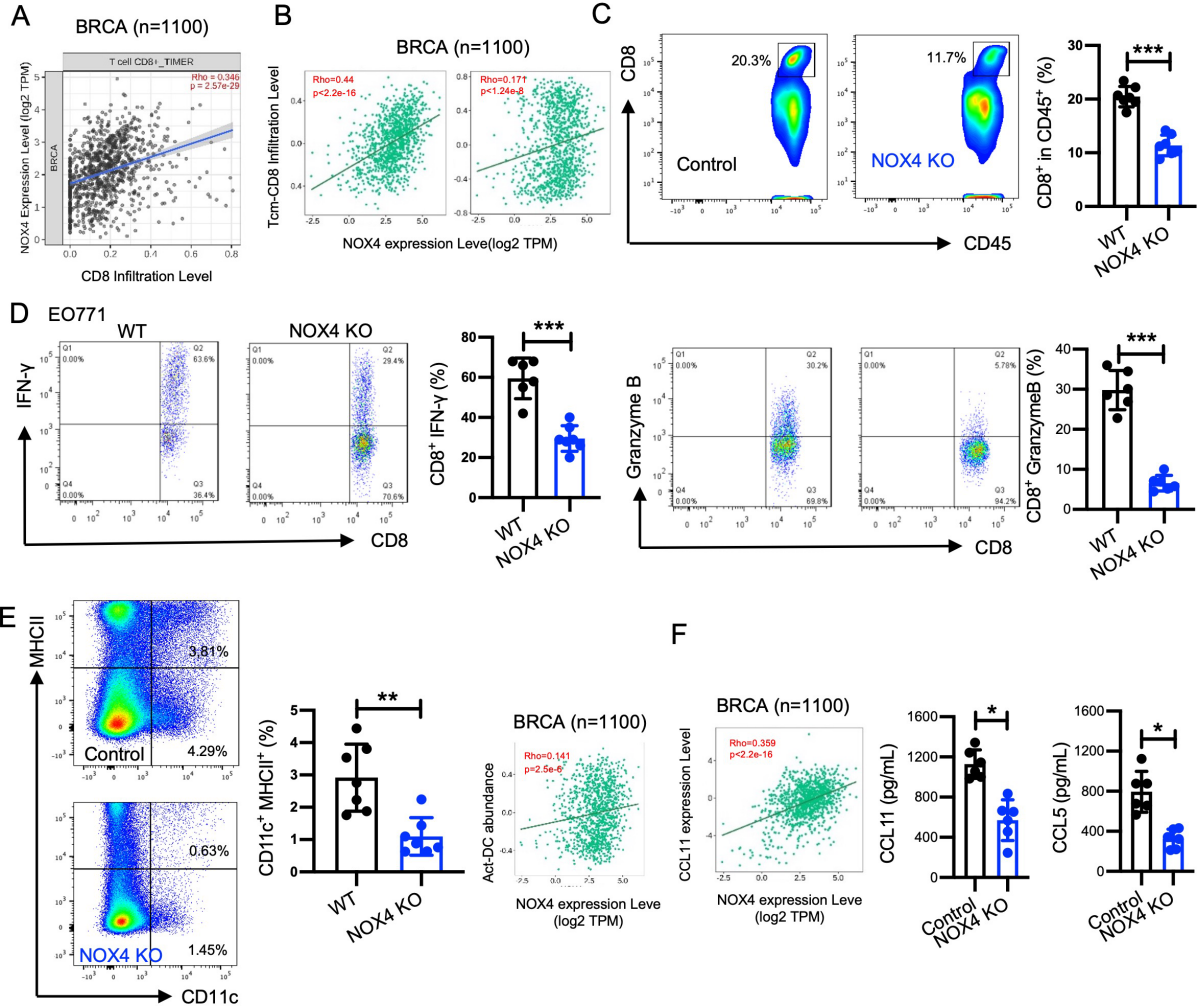
NOX4 enhanced the CD8^+^ T cells mediate antitumor effect. **(A)** TIMER2.0 was used to analysis the correlation between NOX4 expression levels and CD8^+^ T cell infiltration, Scatter plot showing the correlation between NOX4 expression levels and CD8^+^ T cell infiltration in breast cancer (BRCA) samples (n=1100). Rho=0.346, p=2.57e-26. **(B)** TISIDB data: Scatter plot showing the correlation between NOX4 expression and central memory CD8^+^ T cell (Tcm-CD8) infiltration, effector memory CD8+ T cell (Tem-CD8) in BRCA samples (n=1100). Rho=0.44, p<2.2e-16; Rho=0.171, p<1.24e-6. **(C)** Representative flow cytometry plots showing the percentage of CD8^+^ T cells within the CD45^+^ population in EO771 tumors from NOX4 wild-type (WT) and NOX4 knockout (KO) mice. Bar graph quantifying the percentage of CD8^+^ cells, showing a significant reduction in CD8^+^ T cell infiltration in NOX4 KO tumors ***p<0.001. n=6 per group. **(D)** Representative flow cytometry plots showing IFN-γ and Granzyme B expression in CD8^+^ T cells from EO771 tumors of NOX4 WT and NOX4 KO mice. Bar graphs quantifying the percentage of CD8^+^ T cells expressing IFN-γ and Granzyme B, ***p<0.001. n=7 per group. **(E)** Representative flow cytometry plots showing the percentage of DC cells within the EO771 tumors from NOX4 wild-type (WT) and NOX4 knockout (KO) mice. Bar graph quantifying the percentage of DC cells, **p<0.01. n=7 per group. **(F)** TISIDB data: Scatter plot showing the correlation between NOX4 expression and CCL11 expression in BRCA samples (n=1100). A significant positive correlation is observed. Rho=0.359, p<2.2e-16. Bar graph showing the concentration of CCL11, CCL5 in the tumor microenvironment, *p<0.05. Data are presented as mean ± SEM from 3 independent experiments. Statistical significance was determined using two-tailed Student’s t-test.

To investigate the impact of NOX4 on the TME in breast cancer, firstly, we utilized TIMER2.0 to analyze the relationship between NOX4 expression and CD8^+^ T cell infiltration in 1,100 breast cancer cases (25). The analysis, both without distinguishing specific subtypes and by separating subtypes (LumA and LumB, the two most common breast cancer type), consistently demonstrated a positive correlation between NOX4 expression and CD8^+^ T cell infiltration (**Figure 5A**; **Supplementary Figure S4A**). In CD8^+^ T cell subtypes analysis, central memory and effect memory CD8^+^ T cells were positive correlation with NOX4 expression level (**Figure 5B**). This positive correlation indicates that higher levels of NOX4 are associated with increased infiltration of CD8^+^ T cells, particular in central memory CD8^+^ T cells and effector memory CD8^+^ T cells, which are crucial for the anti-tumor immune response, we also observed positive correlation between NOX4 and Th1, Th17, and macrophage (**Supplementary Figure S4B**).

We further validated these findings through animal experiments and flow cytometry to analyze the proportion and function of intertumoral cells. The gating strategy used for flow cytometry analysis is shown in **Supplementary Figure S4C**. NOX4 knockout led to a decreased proportion of CD8^+^ T cells within the tumors (**Figure 5C**). Specifically, the proportion of effector CD8^+^ T cells, which are critical for targeting and destroying cancer cells, was lower in the NOX4 knockout group. These effector CD8^+^ T cells exhibited decreased expression of IFN-γ (**Figure 5D**) and granzyme B (**Figure 5D**), indicating weakened cytotoxic activity. Accordingly, we observed higher levels of DC cells in WT tumors within tumor tissues, and analysis from the TISIDB database also suggests that NOX4 expression is positively correlated with DC cell infiltration (**Figure 5E**). These results suggest that NOX4 knockout not only increases the number of CD8^+^ T cells in the tumor but also enhances their functional capacity.

Although a number of chemokines were able to amplify specific CD8^+^ T-cell or humoral response alone or simultaneously. Increased expression of CXCL9 and CXCL10 has been associated with increased infiltration of activated T cells in many human cancers, including melanoma, ovarian, and colon cancer (26–29). CCL11 and CCL5 was identified as the most potent chemokine in improving immunogenicity, promoting specific CD8 ^+^ T-cell stemness and generating tumor rejection (30, 31). The TISIDB database was used to determine NOX4 expression in different chemokine expression in Breast cancer, the expression of NOX4 is positively correlated with the expression of CCL11(**Figure 5F**). We further assessed the concentration of CCL11 and CCL5 in tumor tissues and found that the NOX4 knockout group exhibited significantly lower levels of CCL11 and CCL5 (**Figure 5F**). This reduction in CCL11 may partly account for the decreased number of CD8+ T cells observed in the tumors of NOX4 knockout mice.

Analysis of CD4^+^ helper T cells indicated no significant changes in the numbers of Th1 cells (IFN-γ^+^), Treg cells (FOXP3^+^), or Th17 cells (IL-17A^+^) following NOX4 knockout (**Supplementary Figure S4D**). This suggests that NOX4 specifically affects CD8^+^ T cells rather than CD4^+^ T cell subsets. Moreover, the levels of TNF-α in myeloid cells and IFN-γ in NK cells were not affected by NOX4 knockout (**Supplementary Figure S4D**), indicating that the impact of NOX4 on the TME is primarily mediated through CD8^+^ T cells.

The results demonstrate that NOX4 significantly affects the TME in breast cancer by modulating CD8^+^ T cell infiltration. The decreased infiltration and weakened functional capacity of CD8^+^ T cells in NOX4 knockout tumors suggest that targeting NOX4 could boost the anti-tumor immune response. However, the combination therapies targeting both NOX4 and CD8^+^ T cells may be necessary to fully exploit this therapeutic strategy.

### Overexpression of NOX4 can improve the prognosis of breast cancer and enhance the efficacy of tumor immunotherapy

**Figure 6:**
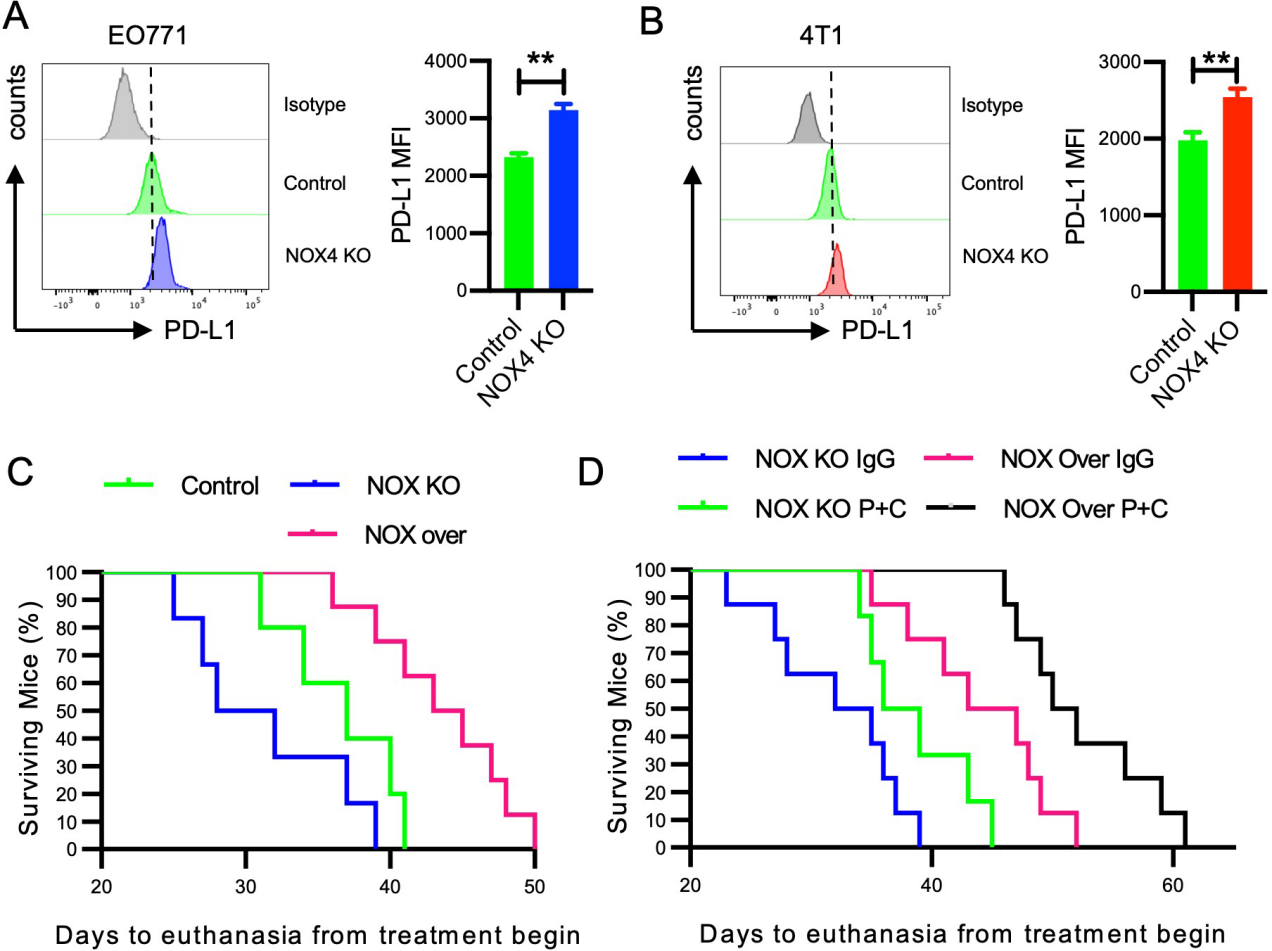
Impact of NOX4 Knockout and Overexpression on PD-L1 Expression and Survival in Mice **(A)** Flow cytometry histograms showing PD-L1 expression in EO771 NOX4 wild-type (Control) and NOX4 knockout (KO) cells. Bar graph quantifying the mean fluorescence intensity (MFI) of PD-L1. **p<0.01. **(B)** Flow cytometry histograms showing PD-L1 expression in 4T1 NOX4 wild-type (Control) and NOX4 knockout (KO) cells. Bar graph quantifying the MFI of PD-L1. **p<0.01. **(C, D)** Kaplan-Meier survival curves showing the percentage of surviving mice over time, following the start of treatment. Different groups include Control, NOX4 knockout (NOX KO), and NOX4 overexpression (NOX over) mice treated with either IgG or a combination of PD-1 and CTLA-4 inhibitors (P+C). n=10 per group. Data are presented as mean ± SEM from 3 independent experiments, Statistical significance was determined using two-tailed Student’s t-test **(A, B)**, or Kaplan-Meier test for survival analysis **(C, D)**.

It is well known that the expression of immune checkpoint molecules by tumor cells plays a major role in shaping the TME (32). We directly measure the expression of PD-L1 in breast cancer cells following NOX4 knockout. The results indicated that NOX4 is a regulatory factor for PD-L1 expression, with NOX4 knockout leading to higher levels of PD-L1 expression in breast cancer cells (**Figures 6A, B**). PD-L1 is an immune checkpoint molecule that inhibits T cell function, allowing cancer cells to evade the immune response. The increased expression of PD-L1 following NOX4 knockout suggests a potential feedback mechanism where the tumor attempts to counteract the enhanced immune infiltration by upregulating PD-L1.

The results of our study strongly indicate the potential value of NOX4 in breast cancer immunotherapy. To further elucidate the role of NOX4, we selected EO771 cells with moderate NOX4 expression and generated NOX4 overexpressing cells through cell transfection. We inoculated three groups of mice with EO771 control cells, NOX4 knockout (KO) cells, and NOX4 overexpressing cells, respectively. The survival curves of the mice were recorded to assess the impact of NOX4 expression on survival outcomes. Our findings demonstrated that mice inoculated with NOX4 overexpressing cells had the longest survival, with an average extension of 8-9 days compared to the control group. In contrast, mice inoculated with NOX4 KO cells exhibited the shortest survival time (**Figure 6C**). This suggests that NOX4 plays a critical role in enhancing the survival of breast cancer-bearing mice, potentially through mechanisms involving immune modulation and tumor suppression.

Given the regulatory role of NOX4 in PD-L1 expression in breast cancer cells and the widespread use of immune checkpoint inhibitor therapy in malignancies, which has significantly improved patient survival, we sought to further investigate the interaction between NOX4 expression and immune checkpoint therapy. Specifically, we compared the survival outcomes of NOX4 KO and NOX4 overexpressing cell lines following treatment with both anti-PD-1 and anti-CTLA-4 antibodies.

The dual-target immunotherapy significantly improved the survival of NOX4 KO mice, indicating that even in the absence of NOX4, immune checkpoint blockade can elicit a beneficial anti-tumor response. However, NOX4 overexpression alone achieved comparable efficacy to that observed with immunotherapy in NOX4 KO mice, suggesting that NOX4 itself may potentiate anti-tumor immunity (**Figure 6C**). Notably, the combination of NOX4 overexpression and dual-antibody immunotherapy produced a synergistic effect, resulting in the longest survival benefit for this group of mice. This synergistic effect implies that NOX4 may enhance the efficacy of immune checkpoint inhibitors, potentially by modulating the tumor microenvironment to favor immune cell infiltration and activation.

## Discussion

Breast cancer is the most common malignancy among women and the second leading cause of cancer-related deaths (33). In 2020, approximately 226,000 new breast cancer cases were reported worldwide, with the highest incidence rates (33). Particularly concerning is triple-negative breast cancer (TNBC), known for its capacity to affect younger women, early metastasis despite optimal adjuvant treatment, and poor prognosis (34).

Our research underscores the crucial role of NOX4 in breast cancer. Our findings demonstrate that knocking out NOX4 in breast cancer cells enhanced the malignant potential of 4T1 and EO771 cells, as evidenced by a increasing their proliferation and migration abilities. In line within HCC (11). Additionally, we discovered that NOX4 knockout reshapes the energy metabolism of breast cancer cells and alters the immune microenvironment, thereby reducing their sensitivity to immunotherapy.

In this study, we utilized CRISPR-Cas9 to establish NOX4 knockout 4T1 and EO771 breast cancer cell line. DNA sequencing and Western blot confirmed the successful establishment of knockout cell lines. Additionally, a NOX4 inhibitor was used to double-confirm the loss of NOX4 function at a functional level. Unlike transient transfection or siRNA-mediated knockdown, this approach effectively avoids potential side effects caused by transfection reagents or ectopic RNA expression (35).

Besides NOX4, other NADPH oxidases like NOX1, NOX2, and NOX5 also contribute to ROS production in tumors, indicating a complex ROS network within the TME (36). Our findings show that NOX4 knockout significantly reduces ROS production in breast cancer cells. However, it does not completely inhibit ROS production. Using NOX4-specific inhibitors significantly reduces ROS levels, highlighting NOX4’s critical role.

Reprogrammed metabolic patterns not only provide additional nutrients necessary for tumor growth but also supply various signals essential for tumor proliferation (37–39). One of the primary pathways through which tumor cells obtain energy is glucose utilization. The main pathway of glucose metabolism in cancer cells is aerobic glycolysis, termed Warburg effect. In cancer cells, glucose uptake and the production of lactate was dramatically increased, even in the presence of oxygen and fully functioning mitochondria (40). This classic type of metabolic change provides substrates required for cancer cell proliferation and division, which is involved in tumor growth, metastatic progression and long-term survival (41, 42). It must be emphasized that both glycolysis and mitochondrial metabolism are crucial to cancer cells in the Warburg Effect (40).

An increase in lipid synthesis is another hallmark of metabolic reprogramming in tumor cells. Enhanced lipid synthesis often results in elevated cholesterol levels within tumor cells (43). Since NOX4 plays a central role in cellular metabolism, our study has observed its significant influence on fatty acid oxidation in breast cancer cells. These findings suggest that NOX4 is involved in the overarching regulation of the cellular metabolic processes. Our research has uncovered that the knockout of NADPH oxidase in breast cancer cells enhances lipid metabolism. This metabolic shift not only provides additional energy for cell growth but also produces key intermediate metabolites, such as acetyl-CoA, which plays a role in regulating PD-L1 expression via induce c-Myc acetylation (44). Fatty acid oxidation (FAO), is a metabolic process that shortens fatty acids by two carbons in each cycle, generating NADH, FADH2, and acetyl-CoA. Acetyl-CoA, the end product of FAO, can be converted back into fatty acids through fatty acid synthesis (FAS). Furthermore, NAD^+^, a product of these reactions, acts as a PD-L1 stabilizer, facilitating tumor immune evasion (45). Cholesterol, derived from acetyl-CoA, is an essential component of biological membranes and a substrate for steroid hormones, with its synthesis being closely linked to enzymes such as acyl-coenzyme A:cholesterol acyltransferase (ACAT) (46).

The oncoprotein MYC is a key regulator of various cellular signaling and metabolic pathways and contributes to drug resistance in breast cancer by enabling cancer cells to reprogram under drug-induced stress (47). MYC-dependent pathways are often elevated in acquired resistance to anti-cancer therapies, making MYC effectors potential targets for treating drug-resistant, MYC-dependent tumors. Identifying factors that regulate MYC expression in breast cancer is crucial. Our found that NOX4 knockout leads to increased MYC levels, and shMYC can reverse the functional changes caused by NOX4 knockout. Although MYC is a broadly acting oncoprotein, the specific downstream molecules mediating these effects are not yet clear, indicating a complex macroscopic regulatory network. Understanding these interactions could offer new strategies for managing drug-resistant breast cancer.

While the increase in CD8^+^ Tcm and Tem cells in the TIDISB dataset does not fully imply the recruitment of tumor antigen-specific T cells, due to it may drive by an inflammatory tumor microenvironment. Our study shows that NOX4, not only suppress the malignant phenotype of 4T1 and EO771 cells but also affects CD8^+^ T infiltration. NOX4 knockout is beneficial to maintain tumor cell phenotype. Additionally, tumor infiltrated CD8^+^ T cells were less activated after NOX4 silencing, suggesting the potential value of combining NOX4 guided signal with immunotherapy to improve clinical outcomes in these tumors. In additional, increased CD8^+^ T cell infiltration may be closely tied to augmented dendritic cell (DC) influx, A likely mechanistic link between NOX4 overexpression, which drives production of reactive oxygen species (ROS), and the resultant pro-inflammatory tumor microenvironment could be key to this enhanced DC infiltration (4, 48). We also observed inconsistencies in Th1, Th17, macrophage populations, between TISIDB analyses and our mouse tumor models. These discrepancies may result from differences in tumor microenvironment dynamics between spontaneous and transplantable tumors, as well as the complete NOX4 knockout in mice compared to the varying NOX4 expression levels analyzed in human data.

Our findings indicate that the upregulation of glycolysis and FAO accompanied by increased PD-L1 expression. This suggests that the regulation of these metabolic pathways could be a mechanism for the upregulation of PD-L1 expression, contributing to tumor immune evasion and progression.

Despite the significant findings of our research, further validation using additional methods such as whole-genome sequencing would be beneficial to rule out any unintended genetic alterations of gene knockout. And our study primarily utilized the mouse model, may not fully recapitulate the complexity and heterogeneity of human breast cancer. Patient-derived xenograft (PDX) models might help to confirm their broader applicability, and the exact mechanism of NOX4 initiate tumor metabolism reprograming and immune regulation need to be further studied. The potential for multiple sources of ROS to offer confounding effects in human disease is a meaningful consideration for therapy development. A greater understanding of the TME’s role in regulating specific immune cells provides scope for developing new approaches and understanding. Work in this arena will broaden the use of checkpoint inhibitors and other immunotherapeutic approaches, helping to select the most appropriate patients and personalize their therapies.

In summary, the results of the present study show that tumor cell NOX4 not only modulates the malignant features of the tumor but also influences the immune microenvironment. Our data suggest that the combination of NOX4 guided signaling and immunotherapy would improve clinical outcomes in these tumors.

## Data Availability

The data presented in the study are deposited in the NCBI repository, accession number PRJNA1218282.
