# Supplementary material for: NOX4 modulates breast cancer progression through cancer cell metabolic reprogramming and CD8+ T cell antitumor activity
Source: Front Immunol. 2025 Feb 7;16:1534936. doi: 10.3389/fimmu.2025.1534936 (PMC11842241; doi:10.3389/fimmu.2025.1534936)
Supplement: Supplementary file 1 [file DataSheet1.docx]

**Supplementary Data**

**NOX4 Modulates Breast Cancer Progression Through Cancer Cell Metabolic Reprogramming and CD8+ T Cell Antitumor Activity**

Yingying Xiong^1^ Yiming Weng^2^ Jian Qin^3^ Jia Feng^2^ Xiaopeng Jing^1^ Chao Luo^4^ Wei Gong^5^ Rui Sun^6^ Min Peng^6^

1. Department of Clinical laboratory, Wuhan Fourth Hospital, Wuhan 430033, China.
2. Cancer Center, Renmin Hospital, Wuhan University, 430060, China.
3. Central Laboratory, Renmin Hospital, Wuhan University, 430060. [qinjian621@163.com](mailto:qinjian621@163.com).
4. Department of Central Laboratory, The Affiliated Huaian No.1 People's Hospital, Nanjing Medical University, 223300, Huai'an, China. [hayylch@njmu.edu.cn](mailto:hayylch@njmu.edu.cn).
5. Department of Oncology, XiangYang Central Hospital, Hubei University of Arts and Science, XiangYang 441021, China. [gongzhewei@sina.com](mailto:gongzhewei@sina.com).
6. Cancer Center, Renmin Hospital, Wuhan University, 430060, China.

**
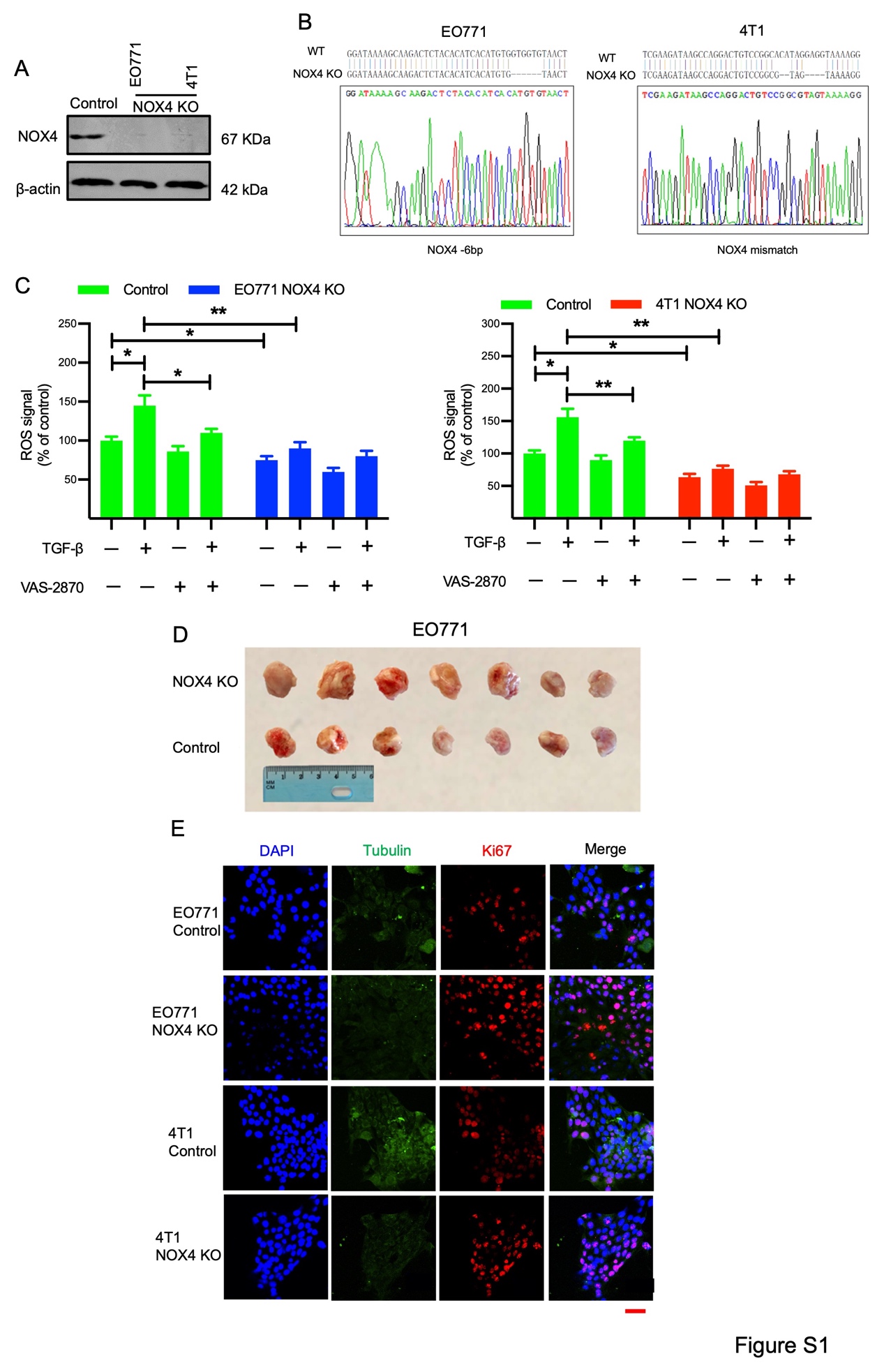
**

**Figure S1. NOX4 Knockout in EO771 and 4T1 Cell Lines and promote tumor growth.**

(A) Western Blot Analysis of NOX4, NOX4 protein expression was detected in EO771 and 4T1 cell lines. Representive data was shown, The NOX4 knockout (KO) cell lines show no detectable NOX4 protein compared to the control. β-actin is used as a control.

(B) The first-generation DNA sequencing was used to validate the NOX4 knockout in EO771 and 4T1 cell lines. The DNA sequencing chromatograms demonstrate the successful knockout of the NOX4 gene, indicated by a 6 bp deletion in EO771 and a mismatch in 4T1.

(C) The relative ROS signal (% of control) in EO771 and 4T1 cell lines, both NOX4 wild-type (Control) and NOX4 knockout (KO), in response to TGF-β treatment and VAS-2870. *p<0.05, **p<0.01. Data is pooled from three independent experiments.

(D) Representative images of tumors formed by EO771 NOX4 KO and control cells. Scale bar is included for size reference. N=7 per group.

(E) Immunofluorescence staining of DAPI (nuclei, blue), Tubulin (green), and Ki67 (proliferation marker, red) in EO771 and 4T1 NOX4 WT and NOX4 KO cells. Representative images were shown.

Data are presented as mean ± SEM from 3 independent experiments. Statistical significance was determined using two-tailed Student’s t-test.


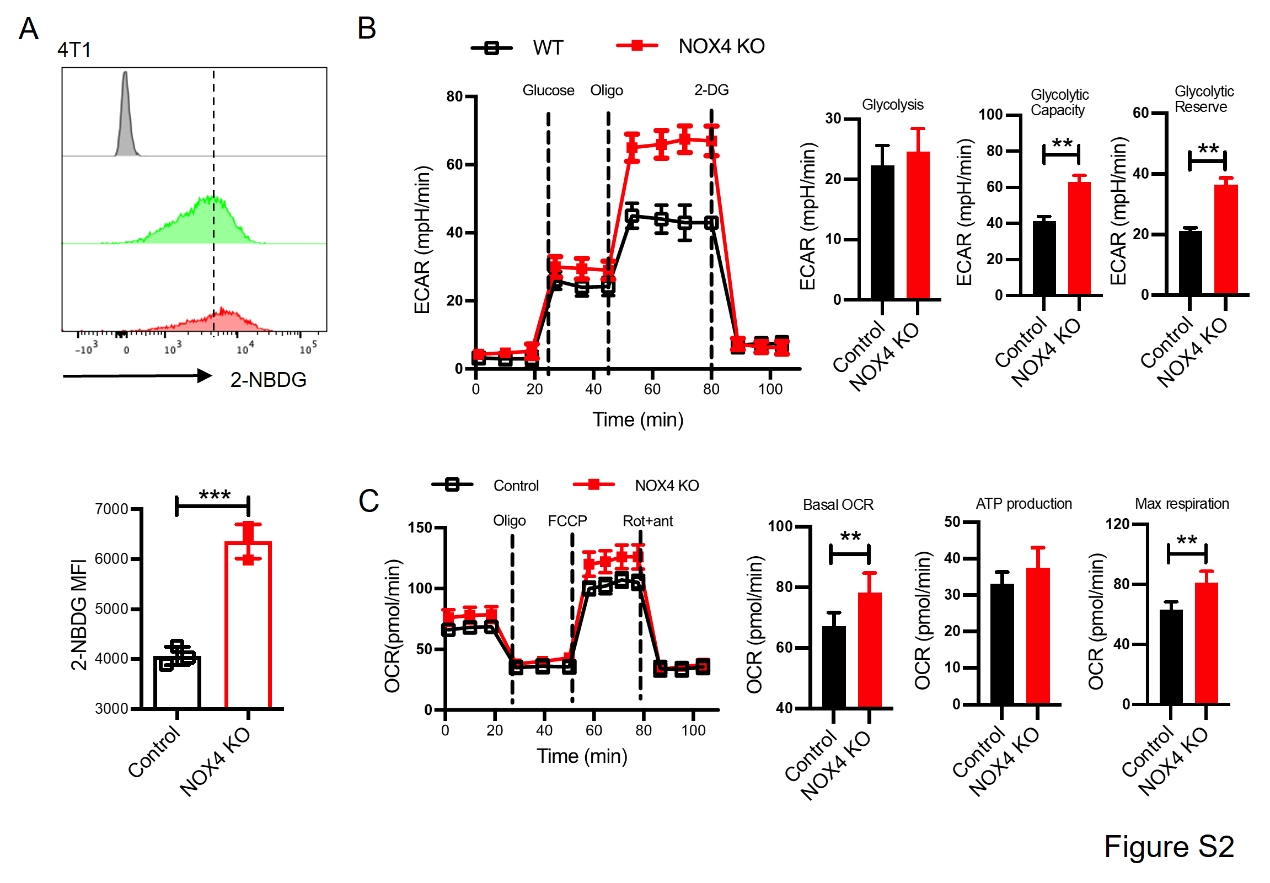


**Figure S2. Metabolic Analysis of NOX4 Knockout in 4T1 Cells**

(A) Flow cytometry histograms showing 2-NBDG uptake in 4T1 NOX4 wild-type (Control) and NOX4 knockout (KO) cells. Bar graph quantifying the mean fluorescence intensity (MFI) of 2-NBDG, indicating increased glucose uptake in NOX4 KO cells ***p<0.001.

(B) Extracellular acidification rate (ECAR) measurements in 4T1 NOX4 WT and NOX4 KO cells over time, following the addition of glucose, oligomycin (Oligo), and 2-deoxyglucose (2-DG). Bar graphs quantifying glycolysis, glycolytic capacity, and glycolytic reserve, **p<0.01. (C) OCR measurements in 4T1 NOX4 WT and NOX4 KO cells over time, following the addition of oligomycin (Oligo), FCCP, and rotenone/antimycin A (Rot+ant).

Bar graphs quantifying basal OCR, ATP production, and maximal respiration, **p<0.01. Note: Data are presented as mean ± SEM from 3 independent experiments. Statistical significance was determined using two-tailed Student’s t-test.


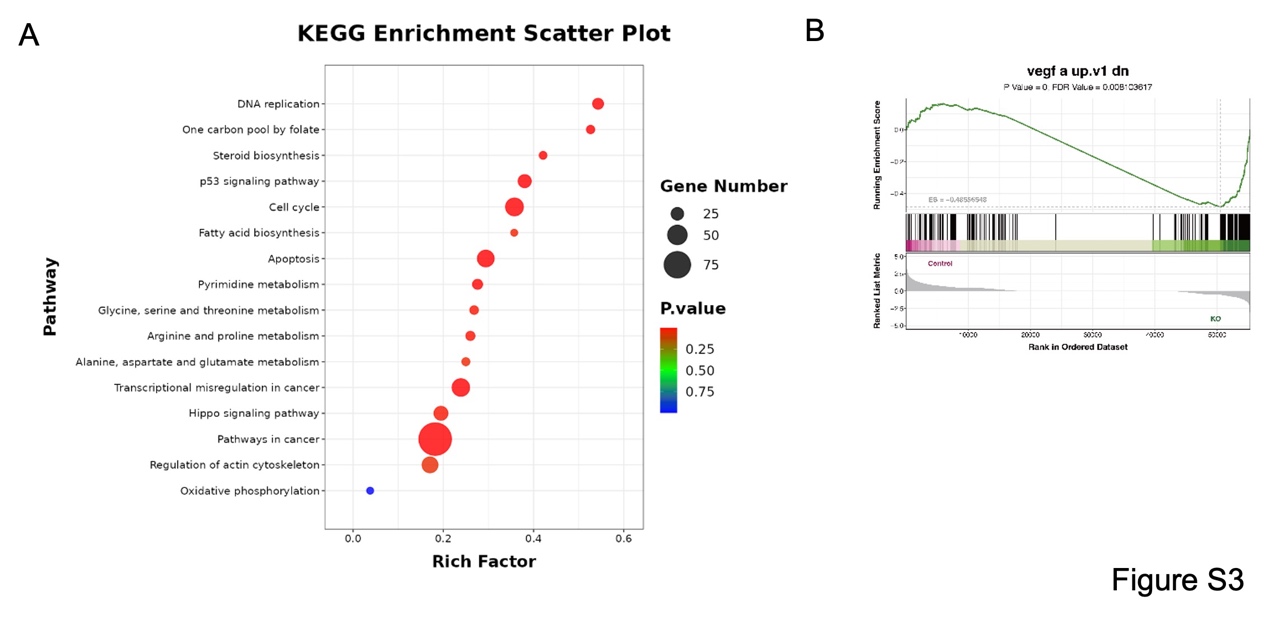


**Figure S3. Pathway Enrichment and Correlation Analysis in Breast Cancer**

(A) KEGG Enrichment Scatter Plot: Bubble plot showing KEGG pathway enrichment analysis of differentially expressed genes in NOX4 knockout EO771 cells. The y-axis lists enriched pathways, while the x-axis shows the rich factor. The size of the bubbles represents the number of genes involved, and the color indicates the p-value, with red being highly significant and blue less so.

(B) GSEA Enrichment of VEGF Signaling Pathway: Gene Set Enrichment Analysis (GSEA) plot for the "vegf a up.v1 dn" gene set in NOX4 knockout cells. The plot shows significant downregulation of VEGF signaling pathway genes in NOX4 knockout cells (p-value = 0, FDR value < 0.01).

**
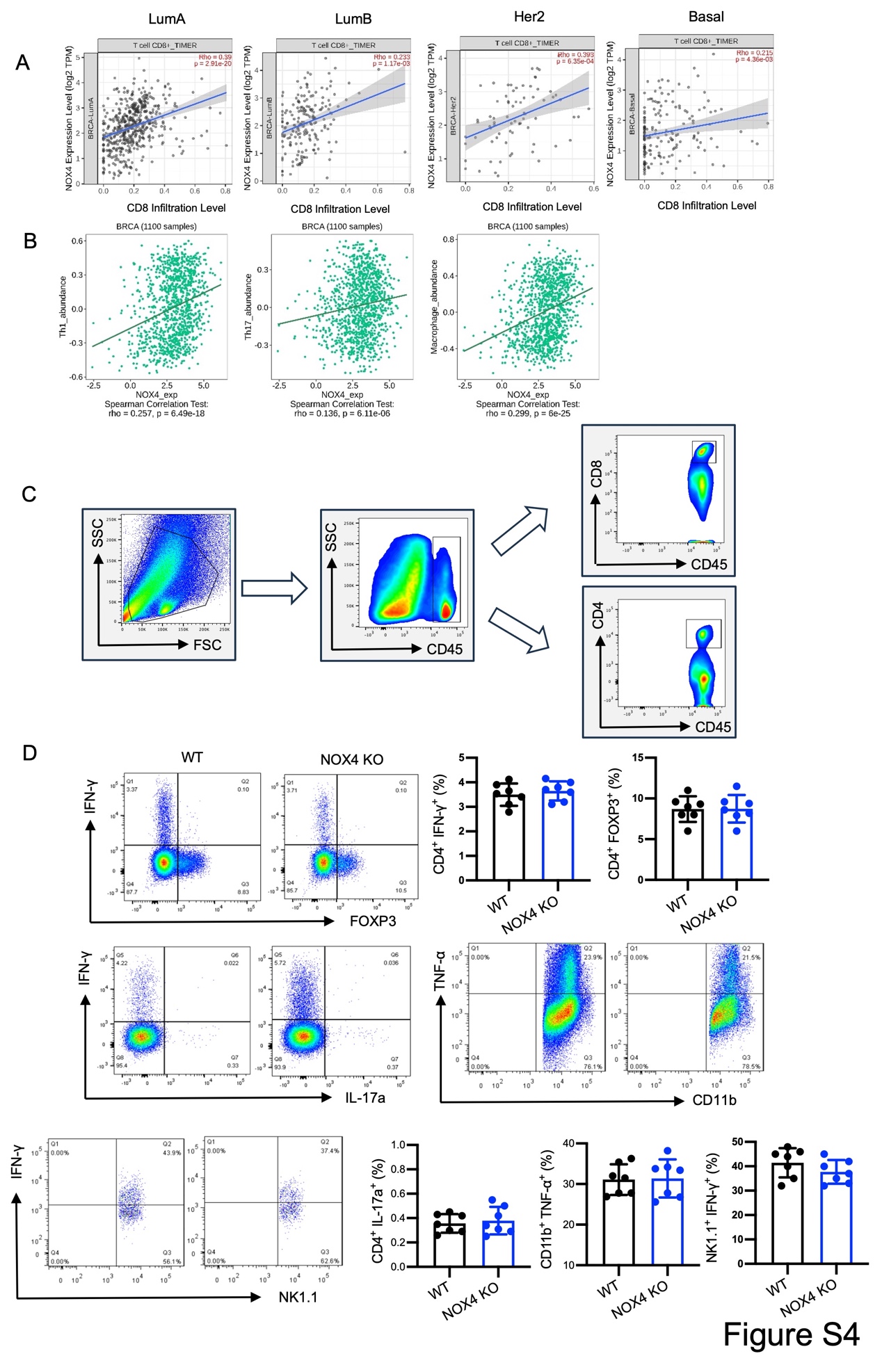
**

**Figure S4. Immune Infiltration in NOX4 Knockout Tumors**

(A) TIMER2.0 data: Scatter plots showing the correlation between NOX4 expression levels and CD8^+^ T cell infiltration in human different breast cancer subtypes (LumA, LumB, Her2, Basal). Significant positive correlations are observed in all subtypes, with correlation coefficients and p-values indicated on each plot.

(B) TISIDB data: Scatter plots showing the correlation between NOX4 expression and the infiltration of Th1, Th17 and macrophage in breast cancer (BRCA) samples (n=1100). Significant positive correlations are observed, with correlation coefficients and p-values indicated on each plot.

(C) Gating Strategy for Flow Cytometry: Schematic representation of the gating strategy used to identify CD8^+^ and CD4^+^ T cells within the CD45^+^ population in tumor samples. Forward scatter (FSC) and side scatter (SSC) plots used to gate on lymphocytes, followed by gating on CD45^+^ cells, and subsequent gating on CD8^+^ and CD4^+^ T cells.

(D) Representative flow cytometry plots showing the expression of IFN-γ and FOXP3 in CD4^+^ T cells, IL-17a in CD4^+^ T cells, TNF-α in CD11b^+^ cells, and IFN-γ in NK1.1^+^ cells from tumors of NOX4 wild-type (WT) and NOX4 knockout (KO) mice. Bar graphs quantifying the percentage of CD4^+^ IFN-γ^+^, CD4^+^ FOXP3^+^, CD4^+^ IL-17a^+^, CD11b^+^ TNF-α^+^, and NK1.1^+^ IFN-γ^+^ cells, showing no significant differences between WT and NOX4 KO tumors for most cell types, but a trend towards reduced IFN-γ and TNF-α expression in some populations, n=7.

Data are presented as mean ± SEM from 3 independent experiments. Statistical significance was determined using two-tailed Student’s t-test.

**Supplemental table 1. Primers used for Real-time PCR**

| **Gene name** | **Forward primer** | **Reverse primer** |
| --- | --- | --- |
| β-Actin | ACGGCCAGGTCATCACTATTC | AGGAAGGCTGGAAAAGAGCC |
| Fh | GAATGGCAAGCCAAAATTCCTT | CGTTCTGTAGCACCTCCAATCTT |
| Aco1 | AGAACCCATTTGCACACCTTG | AGCGTCCGTATCTTGAGTCCT |
| Myc | TTCTACGACTATGACTGCGGA | TGATGGAAGCATAATTCCTGCC |
| CS | GGACAATTTTCCAACCAATCTGC | AGTCAATGGCTCCGATACTGC |
| Idh1 | ATGCAAGGAGATGAAATGACACG | GCATCACGATTCTCTATGCCTAA |
| Ogdh | AGGGCATATCAGATACGAGGG | CTGTGGATGAGATAATGTCAGCG |
| Idh2 | GGAGAAGCCGGTAGTGGAGAT | GGTCTGGTCACGGTTTGGAA |
| Idh3a | TGGGTGTCCAAGGTCTCTC | CTCCCACTGAATAGGTGCTTTG |
| Idh3b | TGGAGAGGTCTCGGAACATCT | AGCCTTGAACACTTCCTTGAC |
| Idh3g | GGTGCTGCAAAGGCAATGC | TATGCCGCCCACCATACTTAG |
| Mdh1 | TTCTGGACGGTGTCCTGATG | TTTCACATTGGCTTTCAGTAGGT |
| Mdh2 | TTGGGCAACCCCTTTCACTC | GCCTTTCACATTTGCTCTGGTC |
| Me1 | GTCGTGCATCTCTCACAGAAG | TGAGGGCAGTTGGTTTTATCTTT |
| Sdha | GGAACACTCCAAAAACAGACCT | CCACCACTGGGTATTGAGTAGAA |
| Sdhb | AATTTGCCATTTACCGATGGGA | AGCATCCAACACCATAGGTCC |
| Sdhaf2 | GCGGTGGTCACCTTGATCC | CCTCTGTAGAAGCGTCTGAATG |
| Sdhc | GCTGCGTTCTTGCTGAGACA | ATCTCCTCCTTAGCTGTGGTT |
| Sdhd | TGGTCAGACCCGCTTATGTG | GGTCCAGTGGAGAGATGCAG |

Uncropped western blot 1.


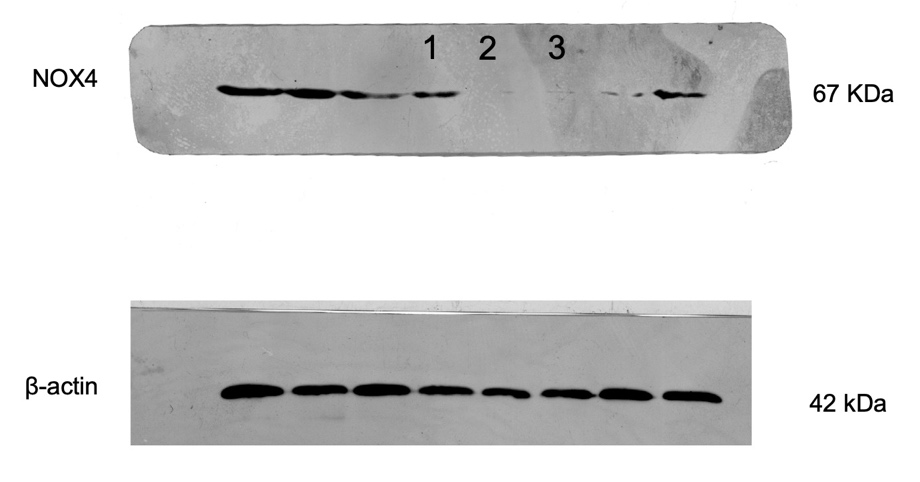


Lane 1, 2 and 3 represent the control, EO771-NOX4 KO and 4T1-NOX4 KO

Uncropped western blot 2.


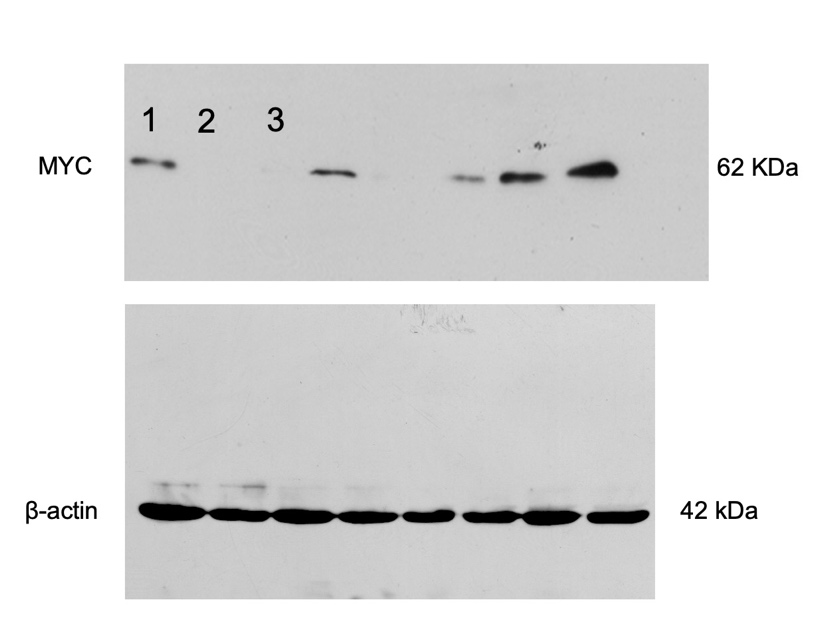


Lane 1, 2 and 3 represent the control, EO771-shMyc and 4T1-shMyc
